# Exosomal NEAT1 from tumor stem cells induces SIRPA^+^ macrophages to enhance immune evasion and glioblastoma progression via upregulating the HSP90B1/STAT3 axis

**DOI:** 10.1186/s13046-026-03729-z

**Published:** 2026-05-11

**Authors:** Nanheng Yin, Zhicheng Zhang, Feiyu Xia, Xiaopei Zhang, Zengyang Li, Tao Zhong, Jiaxin Pan, Geng Liang, Delong Huang, Xiaoxiao Dai, Jun Dong

**Affiliations:** 1 Department of Neurosurgery, The Second Affiliated Hospital of Soochow University, Suzhou, 215004 China; 2 Department of Pathology, The Second Affiliated Hospital of Soochow University, Suzhou, China

**Keywords:** Glioblastoma, Tumor stem cells, Exosomal lncRNA NEAT1, Macrophages

## Abstract

**Background:**

Signal Regulatory Protein Alpha (SIRPA) functions as an inhibitory receptor to suppress phagocytosis of macrophages and promote tumor immune evasion. Recent studies revealed that SIRPA deficiency reprogrammed tumor-associated macrophages toward an antitumor phenotype, and SIRPA functioned independently of CD47. However, the exact role and signaling pathways by which SIRPA^+^ macrophages were induced and exerted their functions in glioblastoma have not been fully elucidated.

**Methods:**

Public single-cell RNA sequencing datasets were analyzed to identify SIRPA^+^ macrophages and characterize their transcriptional states. Immunofluorescence staining was applied to compare difference in SIRPA expression between tumor and peritumoral tissues, and spatial association of SIRPA^+^ macrophages with glioblastoma stem cells (GSCs) was investigated. Phenotypic alterations of macrophages were assessed after coculture with glioblastoma cells or GSCs. Transwell and CCK-8 assays were performed to evaluate the effects of SIRPA^+^ macrophages on proliferation, migration and invasion of tumor cells. CXCL8 secretion by macrophages was quantified with ELISA. Bioinformatic analyses were performed to predict candidate upstream regulators of SIRPA, which were subsequently validated by qPCR, Western blot, RIP, Co-IP, PLA and dual-luciferase assays. Orthotopic xenografts were applied to validate the relevant molecular pathways in vivo.

**Results:**

Bioinformatic analysis of single-cell datasets identified a distinct subset of SIRPA^+^ macrophages, enriched in tumor regions with epithelial–mesenchymal transition (EMT) signatures, correlating with poor clinical prognosis. Immunofluorescence confirmed higher abundance of SIRPA^+^ macrophages within tumor parenchyma and their spatial colocalization with GSC markers. GSC-derived exosomes induced SIRPA expression in macrophages and suppressed their phagocytic ability, thereby enhancing proliferation, migration, invasion and EMT of glioblastoma cells. LncRNA NEAT1 was highly expressed in GSCs and their exosomes. NEAT1 was efficiently transferred into macrophages, then stabilized HSP90B1 and strengthened its interaction with STAT3, thereby enhancing HSP90B1-dependent STAT3 phosphorylation and activating SIRPA transcription. In vivo, knockdown of NEAT1 or SIRPA in macrophages partially reversed intracranial tumor progression induced by GSC-derived exosomes and prolonged survival of tumor-bearing mice, supporting the tumor-promoting role of the NEAT1/HSP90B1/STAT3/SIRPA axis in glioblastoma.

**Conclusions:**

GSC-derived exosomal NEAT1 reprogrammed macrophages toward an immunosuppressive SIRPA^+^ phenotype through the HSP90B1/STAT3 axis, thereby promoting immune evasion and progression of glioblastoma, highlighting NEAT1 as a critical mediator of the crosstalk between GSCs and macrophages, which can serve as a potential therapeutic target against glioblastoma.

**Supplementary Information:**

The online version contains supplementary material available at 10.1186/s13046-026-03729-z.

## Introduction

Glioblastoma, the most common and aggressive primary malignancy of central nervous system (CNS) in adults, accounts for 50.1% of all primary malignant CNS tumors, with an incidence of 4.03 cases per 100,000 persons [1]. Despite maximal resection, standard chemo-radiotherapy with temozolomide and adjuvant treatments, glioblastoma is characterized by rapid progression, extensive infiltration, and inevitable recurrence, resulting in a median survival of less than 15 months [2]. GSCs contribute to therapeutic resistance and possess the capacity to drive recurrence [3]. GSCs play pivotal roles in shaping the immunosuppressive tumor microenvironment (TME) by modulating the phenotypes and functions of immune cells in TME [4, 5]. Therefore, elucidating the molecular mechanisms by which GSCs drive tumor progression and shape a highly immunosuppressive microenvironment is essential for identifying novel therapeutic targets to develop more effective treatment strategies.

Tumor-associated macrophages (TAMs) constitute the major immune-infiltrating population within TME of glioblastoma [6]. Accumulating evidence indicated that TAMs directly interact with glioblastoma cells to promote progression and formation of immunosuppressive TME [7, 8]. Approximately 85% of TAMs are derived from peripheral monocytes (Mo-TAMs), which constitute the dominant population, are mainly localized around the perivascular regions of glioblastoma, and exhibit transcriptional programs distinct from those TAMs are derived from microglia (MG-TAMs) [9–11]. Due to remarkable heterogeneity and plasticity of TAMs within the glioblastoma microenvironment [12, 13], single-cell and spatial transcriptomic technologies have been applied and enabled the investigation of spatially resolved transcriptional programs and cellular interactions of TAMs within segregated glioblastoma niches, revealing multiple TAMs subpopulations with diverse ontogeny, spatial localization, and functional states that cannot be fully captured by the classical M1/M2 paradigm [14, 15], and need further investigations.

Exosomal lncRNAs play crucial roles in remodeling the tumor microenvironment and promoting immunosuppression through intercellular communication between tumor and immune cells [16]. However, the signaling pathways by which GSC-derived exosomal lncRNAs remodeled TAMs and promoted GBM progression have not been fully elucidated.

## Materials and methods

### Bioinformatics analysis

Single-cell RNA-sequencing (scRNA-seq) data from 16 glioblastoma samples were obtained from the GSE182109 dataset. Data preprocessing, quality control, dimensionality reduction, and clustering were conducted with the Seurat package (v4.0) in R. Cells expressing < 200 genes or having mitochondrial genes > 20% were excluded. Principal component analysis (PCA) was applied for initial dimensionality reduction, followed by Uniform Manifold Approximation and Projection (UMAP) for visualization. Cell clusters were annotated based on canonical markers referenced from the CellMarker 2.0 database. The cell communication analysis was performed using the CellChat R package (version 2.1.2). Bulk RNA-seq data and clinical data were retrieved from The Cancer Genome Atlas (TCGA) database.

### Clinical specimens

Glioblastoma tissues, paired peritumoral brain tissues, and blood samples were collected from patients with glioblastoma at the Department of Neurosurgery, the Second Affiliated Hospital of Soochow University. A total of 10 patients newly diagnosed with glioblastoma were included in this study, ranging in age from 45 to 79 years. The pathological diagnosis of glioblastoma was independently confirmed by two experienced pathologists according to the 2021 World Health Organization (WHO) Classification of Tumors of the Central Nervous System (CNS5). This study was approved by the Ethics Committee of the Second Affiliated Hospital of Soochow University (JD-LK-2022-148-01), and written informed consents were obtained from patients or their legal representatives.

### Cell culture and transfection

Human glioblastoma cell lines SNB19, SF295 and U87MG (ATCC, USA) were cultured in Dulbecco’s Modified Eagle Medium (DMEM, Gibco, USA) supplemented with 10% fetal bovine serum (FBS, ScienCell, USA) in a humidified incubator at 37 °C with 5% CO₂. The human monocytic cell line THP-1 (ATCC, USA) was maintained in RPMI-1640 medium (Gibco, USA) containing 10% heat-inactivated FBS. For macrophage induction, THP-1 cells were resuspended at a density of 1 × 10⁶ cells/mL and treated with 100 ng/mL phorbol 12-myristate 13-acetate (PMA, MCE, USA) for 48 h.

Peripheral blood mononuclear cells (PBMCs) were isolated from blood samples of GBM patients. The anticoagulated whole blood was diluted with an equal volume of 1×PBS, gently mixed, and then carefully layered over an equal volume of lymphocyte separation medium (Haoyang, Tianjin, China). The diluted blood was slowly added onto surface of the separation medium and centrifuged at 500 ×g for 30–35 min at room temperature. After centrifugation, the white membrane layer containing PBMCs was carefully collected and transferred to a new centrifuge tube, then 10 mL of 1×PBS was added and the mixture was centrifuged at 300 ×g for 10 min. This washing step was repeated 2 times to remove any residual platelets and separation medium. PBMCs were maintained in RPMI-1640 medium containing 10% heat-inactivated FBS. PBMCs were differentiated into monocyte-derived macrophages (MDMs) by incubation with 100 ng/mL M-CSF for 7 days.

Small interfering RNAs (siRNAs), short hairpin RNAs (shRNAs), overexpression plasmids, and their corresponding negative controls were synthesized from GenePharma (Shanghai, China). Transfections were performed using Lipofectamine 3000 (Invitrogen, USA) according to the manufacturer’s protocol.

### RNA extraction and quantitative reverse transcription–polymerase chain reaction (qRT-PCR)

Total RNA was extracted from cultured cells or tissue samples using TRIzol reagent (Invitrogen, USA) according to the manufacturer’s protocol. For mRNA and lncRNAs detection, reverse transcription was performed using an All-in-One Ultra RT SuperMix (Vazyme, China). Quantitative RT-PCR was conducted using a ChamQ Blue Universal SYBR qPCR Master Mix (Vazyme, China) on an ABI 7500 Real-Time PCR System (Applied Biosystems, USA). GAPDH served as the internal reference gene. Relative gene expression levels were calculated using the 2^^−ΔΔCt^ method. All qPCR reactions were performed in technical triplicates, and each experiment was independently repeated at least three times. Primer sequences are provided in Supplementary Table S1.

### Western blot

Total protein was extracted from cultured cells or tissue samples using RIPA lysis buffer (Beyotime, China) supplemented with protease inhibitor cocktail (Abcam, UK). Protein concentrations were measured using the BCA Protein Assay Kit (Beyotime, China). Equal amounts of protein (20–30 µg) were separated by 10% SDS–polyacrylamide gel electrophoresis (SDS-PAGE) and transferred onto polyvinylidene fluoride (PVDF) membranes (Millipore, USA). After blocking with 5% non-fat milk at room temperature for 2 h, the membranes were incubated overnight at 4 °C with the corresponding primary antibodies. Following washes, membranes were incubated with horseradish peroxidase (HRP)-conjugated secondary antibodies for 2 h at room temperature. Protein bands were visualized with enhanced chemiluminescence (ECL) detection reagents (Fudebio, China) and imaged by a chemiluminescence imaging system (Bio-Rad, USA). Information regarding the antibodies applied was listed in Supplementary Table S2.

### Cell migration and invasion assays

Cell migration and invasion were evaluated in Transwell chambers with 8.0 μm pore size polycarbonate membranes (Corning, USA). For migration assay, glioblastoma cells in the logarithmic growth phase were digested with trypsin, washed, and resuspended in culture medium. A total of 5 × 10⁴ glioblastoma cells in 200 µL medium were seeded into the upper chamber, while 5 × 10⁴ macrophages in 600 µL medium were added to the lower chamber as a chemoattractant. After 48 h of incubation at 37 °C in a humidified incubator with 5% CO₂, non-migrated cells on the upper surface of the membrane were gently removed with a cotton swab. Migrated cells on the lower surface were fixed with 4% paraformaldehyde for 20 min at room temperature, stained with 1% crystal violet, and counted under a light microscope. For invasion assay, the upper chamber was pre-coated with Matrigel (Corning, USA) diluted 1:8 in medium and incubated at 37 °C for solidification. The subsequent steps were identical to those used in the migration assay.

### Cell Counting Kit-8 (CCK-8) assay

Cell proliferation was assessed using the Cell Counting Kit-8 (CCK-8, Dojindo, Japan). Glioblastoma cells were seeded into 96-well plates at a density of 3 × 10³ cells per well in 100 µL of DMEM supplemented with 10% FBS. Wells containing only medium and CCK-8 reagent served as blank control. At the indicated time points (every 24 h), 10 µL of CCK-8 solution was added to each well and incubated at 37 °C for 2 h. The absorbance at 450 nm was measured with a microplate reader (Tecan, Switzerland). All experiments were performed in triplicate.

### In vitro phagocytosis assay

THP-1 monocytes were labeled with 5 µM DiD dye (MCE, USA) in serum-free RPMI-1640 medium and incubated at 37 °C for 2 h in the dark. After labeling, the cells were washed with phosphate-buffered saline (PBS; Gibco, USA), resuspended in RPMI-1640 medium with 10% FBS, and seeded at a density of 5 × 10⁴ cells per 35 mm glass-bottom dish (Nest, China). Glioblastoma cells were digested with trypsin, centrifuged, and resuspended in serum-free medium. Then cells were labeled with 1 µL CFSE (MCE, USA) at 37 °C for 30 min in the dark. After labeling, cells were washed three times with PBS and collected by centrifugation. Subsequently, 5 × 10⁴ CFSE-labeled tumor cells were added to each dish containing DiD-labeled macrophages. After 4 h of co-incubation at 37 °C in 5% CO₂, phagocytic activity was evaluated by confocal laser scanning microscopy (Zeiss, Germany).

### Flow cytometry

To quantify the phagocytic efficiency of macrophages against glioblastoma cells, glioblastoma cells were first digested with trypsin, centrifuged, and resuspended in serum-free medium. Tumor cells were then labeled with 1 µL CFSE (MCE, USA) at 37 °C for 30 min in the dark. After labeling, cells were washed three times with PBS and collected by centrifugation. A total of 5 × 10⁴ CFSE-labeled glioblastoma cells were co-cultured with an equal number of THP-1-derived macrophages in 24-well plates for 4 h at 37 °C in a humidified incubator containing 5% CO₂. Following co-incubation, cells were harvested using trypsin, washed three times with PBS, and stained with anti-CD11b antibody (BioLegend, USA) for 20 min at room temperature in the dark. Subsequently, glioblastoma cells and macrophages were washed again with PBS and resuspended for analysis. Fluorescence was detected with the Navios Flow Cytometer (Beckman Coulter, USA), and data were analyzed with FlowJo software. Phagocytic efficiency was calculated as the percentage of CD11b⁺CFSE⁺ double-positive cells among the total macrophage population.

### Isolation and purification of exosomes

Exosomes were isolated from the conditioned medium of glioblastoma stem-like cells GSC11, GSC23 (MD Anderson Cancer Center, USA) and normal human astrocytes (NHAs). GSCs were cultured in DMEM/F12 medium supplemented with 20 ng/mL basic fibroblast growth factor (bFGF) and 20 ng/mL epidermal growth factor (EGF) for 4 days. NHAs were maintained in DMEM medium containing 10% exosome-depleted FBS under standard conditions (5% CO₂, 37 °C) for 48 h. The culture medium was collected, sequentially centrifuged at 2,000 × g for 30 min to remove dead cells, followed by 12,000 × g for 45 min at 4 °C to eliminate cellular debris and large vesicles. The clarified supernatant was then subjected to ultracentrifugation at 100,000 × g for 70 min at 4 °C. The resulting exosome pellets were gently resuspended in 50–100 µL sterile PBS and stored at − 80 °C for subsequent experiments. Exosomal proteins were quantified with BCA Protein Assay Kit (Beyotime, China). For functional assays, the final concentration of exosomes in the cell culture medium was 30 µg/mL.

### Transmission Electron Microscopy (TEM) and Nanoparticle Tracking Analysis (NTA)

Prior to TEM and NTA analysis, exosome samples were diluted into 10 µg/µL with PBS. Briefly, 10 µL of exosome sample was placed onto copper grids and fixed with 4% paraformaldehyde for 10 min at room temperature. After fixation, the grids were washed three times with PBS to remove excess paraformaldehyde. The grids were then negatively stained with 2% uranyl acetate solution for 5 min and air dried. The samples were visualized under a transmission electron microscope (Hitachi HT-7700, Japan) at an accelerating voltage of 80 kV to assess the ultrastructural morphology of purified exosomes.

For size distribution and concentration analysis, Nanoparticle Tracking Analysis (NTA) was performed using the ZetaView PMX 110 system (Particle Metrix, Germany). Prior to measurement, the instrument was calibrated by diluting a 100 nm polystyrene bead standard solution 250,000-fold with ultrapure water, and 1 mL of the diluted standard solution was used for automatic calibration. After calibration, the sample chamber was cleaned with 1× PBS buffer. Exosome samples were then diluted 1000-fold, achieving a final concentration of approximately 10⁷ particles/mL. NTA measurements were performed according to the manufacturer’s protocols, and particle size distribution and concentration were determined by analyzing the Brownian motion of the exosomes in solution.

### Identification of exosome endocytosis

Purified exosomes were labeled with DiI fluorescent dye (MCE, USA). Briefly, 10 µg of exosomes were incubated with DiI dye at room temperature for 5 min in the dark. The labeling reaction was terminated, and free dye was removed by ultracentrifugation at 100,000 ×g for 70 min at 4 °C. The exosome pellet was resuspended in PBS for subsequent experiments.

DiI-labeled exosomes (30 µg/mL) were added to culture medium of THP-1-derived macrophages and incubated with macrophages at 37 °C for 8 h. Then macrophages were subsequently washed with PBS and fixed with 4% paraformaldehyde for 20 min, and visualized with confocal laser scanning microscopy (Zeiss, Germany).

### RNA Immunoprecipitation (RIP) assay

RNA immunoprecipitation was performed with Magna RIP™ RNA-Binding Protein Immunoprecipitation Kit (Millipore, USA) according to the manufacturer’s protocols. Briefly, magnetic beads were incubated with specific antibody at room temperature for 25 min to allow antibody binding. Subsequently, antibody-coated beads were mixed with cell lysates and incubated overnight at 4 °C with gentle rotation to capture RNA-protein complexes, then treated with proteinase K at 55 °C for 1 h to digest proteins and release the bound RNA. RNA was extracted with phenol : chloroform : isoamyl alcohol (125:24:1) and purified for downstream analysis. The enrichment of target RNAs was assessed by qRT-PCR.

### Immunofluorescence (IF) and Hematoxylin–Eosin (HE) staining

Paraffin-embedded tissue samples were sectioned at thickness of 4 μm. For immunofluorescence analysis, tissue sections were dewaxed in xylene and rehydrated through a graded ethanol series. Endogenous peroxidase activity was blocked by incubating sections with 3% hydrogen peroxide for 10 min at room temperature. Sections were then incubated with goat serum for 20 min to block nonspecific binding.

Primary antibodies were applied and incubated overnight at 4 °C. Sections were washed and incubated with fluorophore-conjugated secondary antibodies for 2 h at room temperature. Nuclei were counterstained with DAPI. Fluorescence images were acquired under confocal microscopic view. For HE staining, tissue sections were stained with hematoxylin for 5 min, followed by differentiation, bluing, eosin counterstaining, dehydration, and mounting with neutral resin. Stained sections were visualized under light microscope. For cell immunofluorescence, cultured cells grown on glass coverslips were fixed with 4% paraformaldehyde for 20 min, permeabilized with 0.2% Triton X-100, and blocked with 5% bovine serum albumin (BSA). Cells were then incubated with antibodies, followed by nuclear staining with DAPI and fluorescence imaging.

### Dual-luciferase reporter assay

The potential binding sites of upstream transcription factor STAT3 in the promoter region of SIRPA were predicted with bioinformatic analysis via UCSC Genome Browser and the JASPAR database, then verified with Dual-Luciferase Reporter Assay. Briefly, three truncated fragments of the SIRPA promoter (designated as region 1, region 2, and region 3) were cloned into the pGL4.10 luciferase reporter vector (Promega, USA). HEK-293T cells were seeded in 24-well plates and co-transfected with the corresponding luciferase constructs, pCDNA3.1-STAT3 expression vector, and Renilla luciferase control plasmid (pRL-TK), respectively, with Lipofectamine 3000 (Invitrogen, USA), according to the manufacturer’s instructions. After 48 h of transfection, cells were lysed and luciferase activity was analyzed with Dual-Luciferase Reporter Assay System (Promega, USA). Firefly luciferase activity was normalized to Renilla luciferase activity to control for transfection efficiency. Luminescence was detected with microplate reader (Tecan, Switzerland).

### Cycloheximide (CHX) chase assay

To evaluate protein stability, macrophages were transfected with either control siRNA or NEAT1 siRNA with Lipofectamine 3000 (Invitrogen, USA) according to the manufacturer’s instructions. After 48 h of transfection, macrophages were treated with 100 µM cycloheximide (CHX, MCE, USA) to inhibit de novo protein synthesis. Macrophages were then harvested at 0, 3, 6, 9, and 12 h following CHX treatment. Protein expression was analyzed by Western blotting and identified with an anti-HSP90B1 monoclonal antibody (Proteintech, China).

### Protein degradation assay

To investigate protein degradation via the ubiquitin–proteasome pathway, THP-1 derived macrophages were transfected with the indicated control siRNA or NEAT1 siRNA with Lipofectamine 3000 (Invitrogen, USA) according to the manufacturer’s instructions. After 48 h, cells were treated with 10 μM of proteasome inhibitor MG132 (MCE, USA) and incubated for 8 h, then were lysed in RIPA buffer supplemented with protease inhibitors. Equal amounts of protein lysates were incubated with anti-HSP90B1 antibody and Protein A/G magnetic beads (Life Technologies, USA) overnight at 4 °C with gentle rotation. The immunoprecipitates were washed and boiled in SDS sample buffer, then Western blotting to detect polyubiquitinated HSP90B1 with anti-ubiquitin antibody (CST, USA).

### Enzyme-Linked Immunosorbent Assay (ELISA)

The culture supernatants of macrophages were collected 48 h following the indicated treatments. The concentration of C-X-C motif chemokine ligand 8 (CXCL8) in supernatant was measured with ELISA kit (Boster, China) in accordance with the manufacturer’s protocols.

### Co-Immunoprecipitation (Co-IP)

Cells were harvested and lysed in ice-cold IP lysis buffer (Thermo Fisher Scientific, USA) supplemented with protease and phosphatase inhibitor cocktails. The lysates were incubated on ice for 30 min and centrifuged at 14,000 × g for 15 min at 4 °C to remove cell debris. The supernatant was collected and precleared with control IgG and Protein A/G magnetic beads (Life Technologies, USA) for 1 h at 4 °C. Subsequently, equal amounts of proteins were incubated with the specific primary antibody overnight at 4 °C with gentle rotation. Immune complexes were then captured using fresh Protein A/G magnetic beads for an additional 2 h at 4 °C. After extensive washing with ice-cold IP wash buffer, the immunoprecipitates were eluted by boiling in SDS sample buffer and analyzed by Western blot with the indicated antibodies.

### Proximity Ligation Assays (PLAs)

PLAs were performed with Duolink PLA Probemaker Kit (Sigma-Aldrich, USA) according to the manufacturer’s instructions. Rabbit anti-STAT3 (CST, USA) and rabbit anti-HSP90B1 (Proteintech, China) antibodies were directly conjugated with PLUS and MINUS oligonucleotide probes, respectively. The rabbit anti-STAT3–PLUS probe and rabbit anti-HSP90B1–MINUS probe were applied at dilutions of 1:5 and 1:10, respectively. Macrophages were fixed with 4% paraformaldehyde (PFA) and permeabilized with 0.25% Triton X-100 in PBS prior to antibody incubation. After the ligation and amplification, samples were counterstained with DAPI and visualized under a confocal laser scanning microscope (Zeiss, Germany).

### In vivo xenograft model

Female BALB/c nude mice (4 weeks old, 15–20 g) were randomly assigned to three groups. A total of 5 × 10⁵ U87 MG-Luci cells were mixed with 1 × 10⁵ conditioned macrophages at a 5:1 ratio and stereotactically injected into the right caudate nucleus of each mouse brain via a micro-burr hole (2 mm lateral to the bregma, 3 mm in depth) under general anesthesia with 0.3% pentobarbital sodium administered intraperitoneally. The conditioned macrophages in the three groups were treated with PBS and transfected with control shRNA (sh-NC&PBS), pretreated with GSC23-derived exosomes and transfected with control shRNA (sh-NC&GSC23-exo), or pretreated with GSC23-derived exosomes and transfected with shRNA targeting SIRPA (sh-SIRPA&GSC23-exo), respectively. Intracranial tumor development was monitored at designated time points via bioluminescence imaging system.

To further determine the role of GSC-derived exosomal NEAT1 in modulating SIRPA⁺ macrophages and promoting glioblastoma progression, a parallel experimental design was adopted. U87MG-Luci cells were co-injected with macrophages either treated with PBS and transfected with shRNA as negative control (sh-NC&PBS), or macrophages pretreated with GSC23-derived exosomes and transfected with shRNA negative control (sh-NC&GSC23-exo), or macrophages pretreated with GSC23-derived exosomes and transfected with shRNA targeting NEAT1 (sh-NEAT1&GSC23-exo), respectively. All animal experiments were approved by the Institutional Animal Care and Use Committee of Soochow University (SUDA20210708A03) and conducted in compliance with institutional ethical guidelines.

### Statistical analysis

All statistical analyses were performed using GraphPad Prism software (version 9.3, GraphPad Software, USA). Data are expressed as mean ± standard deviation (SD) from at least three independent experiments. Comparisons between two groups were assessed using unpaired two-tailed Student’s t-test. For comparisons among more than two groups, one-way analysis of variance (ANOVA) followed by appropriate post hoc tests was applied. F-tests were performed to assess the homogeneity of variances. Survival curves were generated using the Kaplan–Meier method and compared using the log-rank test. A p-value less than 0.05 was considered statistically significant (*p < 0.05; **p < 0.01; ***p < 0.001; ****p < 0.0001). *p* > 0.05 was considered not significant and denoted as “ns” (not significant).

## Results

### Tumor-specific SIRPA^+^ TAMs were identified by scRNA-seq in glioblastoma datasets

**Fig. 1:**
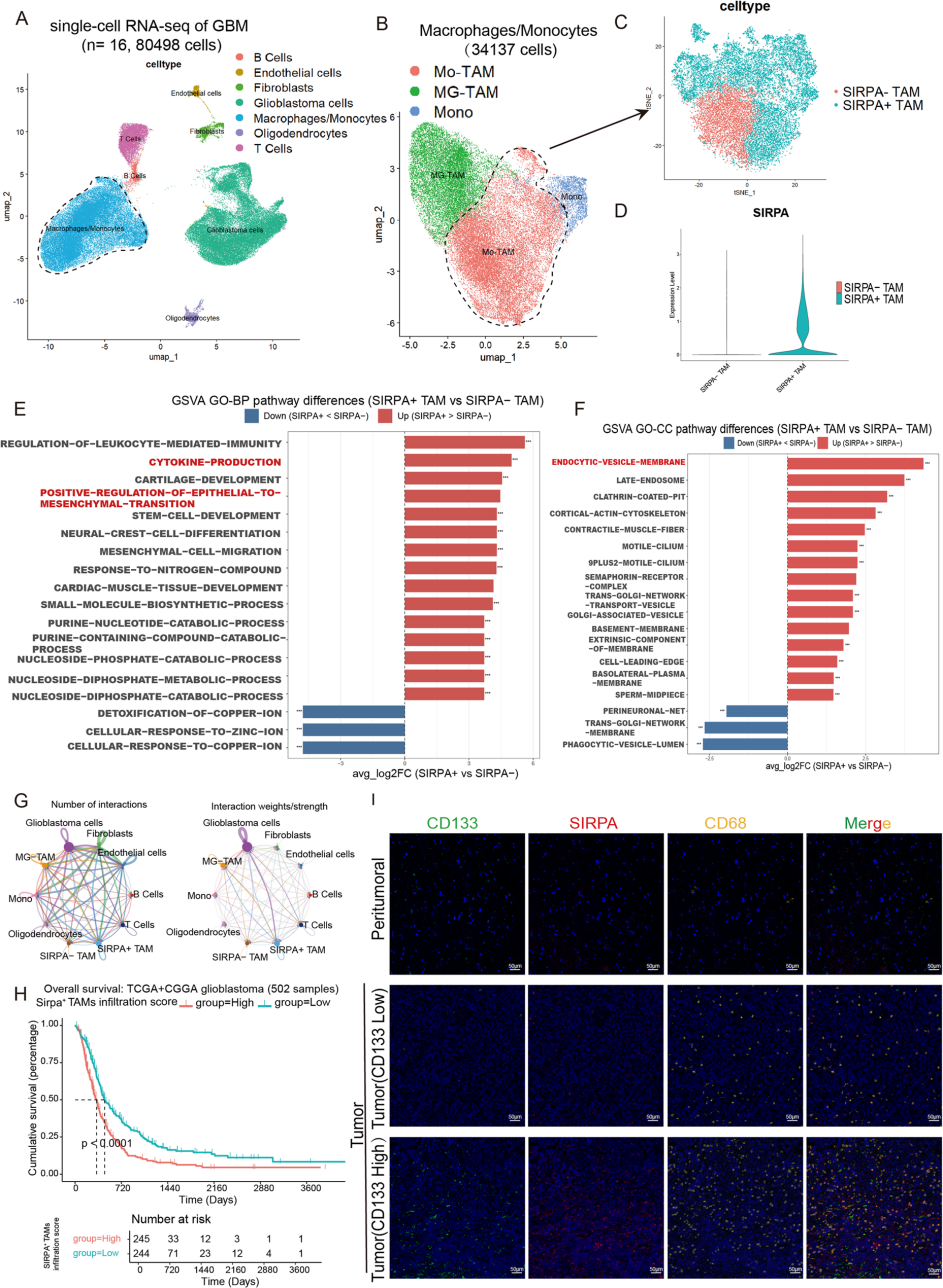
Tumor-specific SIRPA⁺ TAMs were positively associated with GSCs and poor prognosis in glioblastoma. **A** UMAP plot of seven major cell types identified from single-cell RNA sequencing of 16 glioblastoma samples. **B** UMAP plot illustrating distinct monocytes, MG-TAMs, and Mo-TAMs clusters identified from single-cell RNA-seq datasets of glioblastoma. **C** tSNE plot showing six distinct Mo-TAM clusters. **D** violin plot displaying differential SIRPA expression across clusters. **E-F** GSVA-based differential pathway enrichment analysis between SIRPA⁺ and SIRPA⁻ TAMs. **G** CellChat analysis depicting intercellular communications between SIRPA⁺/SIRPA⁻ TAMs and other cell populations. **H** Kaplan–Meier analysis of overall survival based on the SIRPA⁺ TAM infiltration score in 502 glioblastoma samples from the TCGA and CGGA cohorts. **I** Representative immunofluorescence images showing SIRPA (red), CD68 (yellow), CD133⁺ GSCs (green), and DAPI (blue) staining in tumor and peritumoral tissues of glioblastoma patients

Single-cell RNA sequencing data of glioblastoma (GSE182109) obtained from the GEO database was analyzed to determine the cellular composition of glioblastoma. After quality control and cell doublet filtration (Fig. S1A), the transcriptomes of 80,498 cells were retained for subsequent analysis. Following dimensionality reduction with Uniform Manifold Approximation and Projection (UMAP), 18 clusters were identified and subsequently annotated into seven major cell types as follows: glioblastoma cells (*n* = 33926), Macrophages/Monocytes (*n* = 34137), T cells (*n* = 6809), B cells (*n* = 1378), endothelial cells (*n* = 846), fibroblasts (*n* = 1757), oligodendrocytes (*n* = 1645, Fig. 1A and S1B-J). Macrophages/Monocytes were further clustered into monocytes, monocyte-derived tumor-associated macrophages (Mo-TAMs), and microglia-derived TAMs (MG-TAMs) based on canonical cell type markers (Fig. 1B and S2A-B). To determine the heterogeneity of Mo-TAM clusters in diffuse glioblastoma, Mo-TAMs were re-clustered and identified two distinct subpopulations: SIRPA⁺ TAMs and SIRPA⁻ TAMs (Fig. 1C–D and Fig. S2C-D).

### SIRPA^+^ TAMs are positively associated with GSCs quantity and poor glioblastoma prognosis

Gene set variation analysis (GSVA) revealed that SIRPA⁺ TAMs were enriched in gene signatures related to cytokine production, regulation of EMT, and endocytic vesicle formation, compared with SIRPA⁻ TAMs (Fig. 1E-F and Fig. S2E-F). CellChat analysis further revealed that SIRPA⁺ TAMs displayed stronger and more extensive intercellular communication with glioblastoma cells than SIRPA⁻ TAMs (Fig. 1G and Fig. S2G). Kaplan–Meier survival analysis demonstrated that glioblastoma patients with higher infiltration of SIRPA⁺ TAMs had significantly poorer overall survival (Fig. 1H). Besides, immunofluorescence staining of glioblastoma and peritumoral tissues showed that the expression of CD133 (marker of GSCs) and SIRPA increased in tumor regions compared with adjacent peritumoral tissue. Notably, tumors with high CD133 expression also exhibited increased SIRPA expression, suggesting a potential correlation between SIRPA⁺ TAM infiltration and GSCs abundance (Fig. 1I). Collectively, these results disclosed that SIRPA⁺ TAMs were enriched in parenchyma of glioblastoma tissues and co-existence with GSCs can be observed.

### GSCs promoted transformation of SIRPA^+^ TAMs via exosomal signaling

**Fig. 2:**
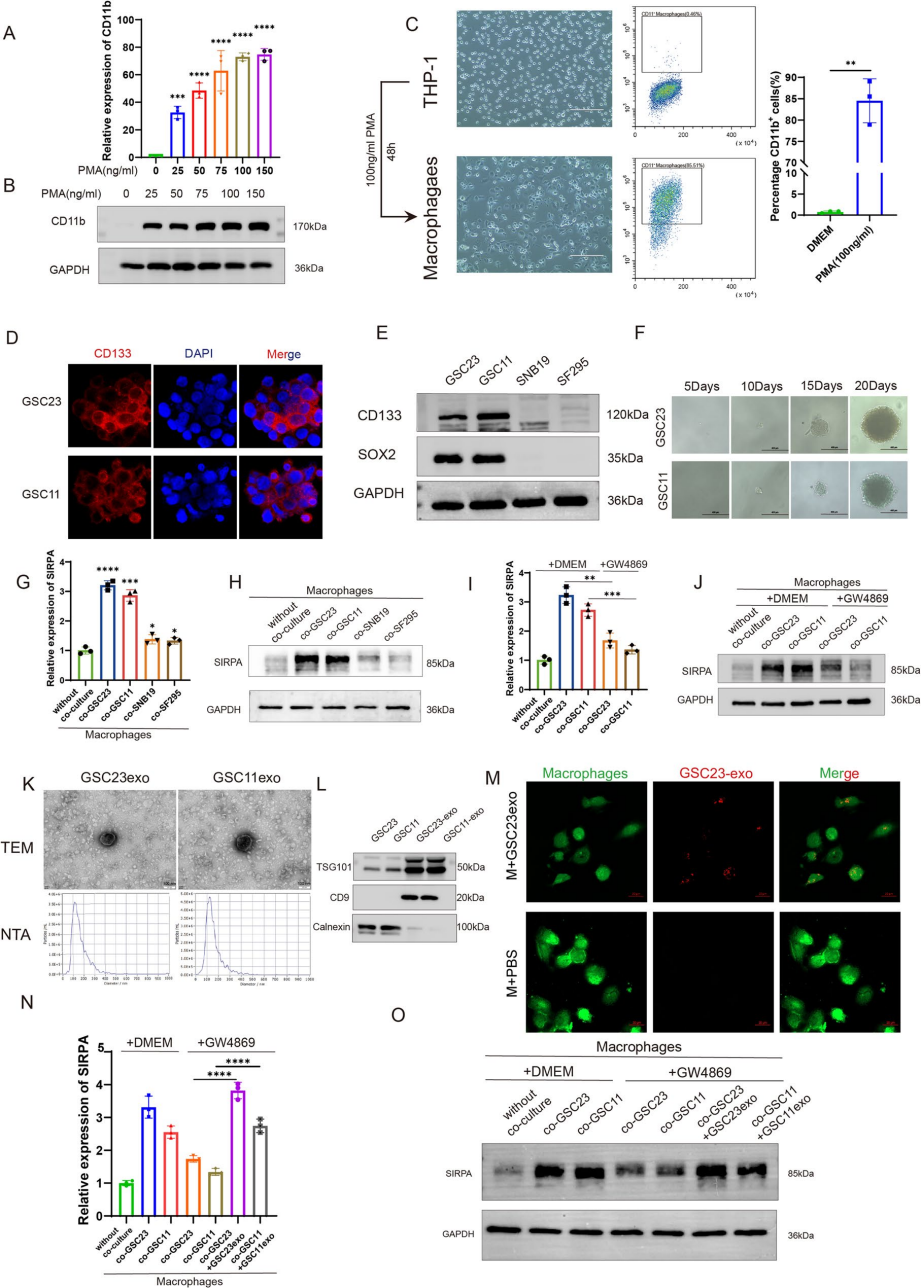
GSCs-exos promoted transformation of THP-1-derived macrophages into SIRPA^+^ TAMs. **A**-**B** Western blot and qPCR disclosed that PMA induced THP-1 differentiation into macrophages in a dose-dependent manner. **C** Differentiation of macrophage was confirmed by characteristic morphological changes and expression of macrophage surface marker CD11b with flow cytometry. **D**-**E** Verification of surface markers of GSCs in GSC23 and GSC11 cells by immunofluorescence and Western blot. **F** ELDA assay confirmed the self-renewal capacity of GSC23 and GSC11 cells. **G**-**H** Western blot and qPCR analyses demonstrated that coculture with GSC23 or GSC11 cells significantly upregulated SIRPA in macrophages. **I**-**J** Addition of GW4869 reversed SIRPA upregulation of macrophages induced by coculture with GSC23 or GSC11 cells, as confirmed by Western blot and qPCR analyses. **K** Representative TEM images of exosomes and size-distribution profile measured by NTA. **L** Exosomes markers detected by Western blot. **M** Representative fluorescence images showing the engulfment of GSC23-exos by macrophages. **N**-**O** Western blot and qPCR analyses showing SIRPA downregulation of macrophages after addition of GW4869 was reversed by supplementation with GSC23-exos or GSC11-exos

THP-1 derived macrophages were induced after PMA stimulation, with marked upregulation of CD11b expression (Fig. 2A–C). GSCs expressed high level of stemness markers CD133 and SOX2, exhibited self-renewal capacity as well (Fig. 2D–F). Indirect Transwell co-culture system was established between macrophages and GBM cells or GSCs. Macrophages cultured without co-culture with any other cells were applied as the control group. Compared with co-culture with glioblastoma cells (SNB19 and SF295), co-culture with GSCs (GSC23 and GSC11) markedly increased SIRPA expression in THP-1-derived macrophages (Fig. 2G–H). Both the enrichment of extracellular vesicle endocytosis pathways in SIRPA⁺ TAMs and the physical separation of GSCs from macrophages in the co-culture system disclosed that GSCs induced SIRPA⁺ TAM polarization via exosome-mediated signaling. GW4869 was a widely used inhibitor against exosome secretion [17]. The concentration of exosomes isolated from supernatant of GSCs after GW4869 treatment was analyzed with BCA assay, which disclosed that GW4869 suppressed exosome secretion from GSCs significantly (Fig. S3A). Western blot analysis further confirmed that GW4869 did not affect SIRPA expression in macrophages (Fig. S3B). To determine whether GSCs induce SIRPA⁺ TAMs through exosome secretion, GSC-derived exosome release was inhibited with addition of GW4869, which demonstrated that GW4869 significantly reduced the ability of GSCs to induce SIRPA expression in macrophages (Fig. 2I–J).

Subsequently, exosomes were isolated from the supernatant of GSCs cultures by ultracentrifugation. TEM and NTA confirmed that the isolated vesicles were exosome-like structures with diameters ranging from 30 to 150 nm (Fig. 2K). SIRPA expression was evaluated in macrophages after supplementation with 0, 5, 10, 20, 30, or 40 µg/mL GSC-derived exosomes, respectively for 48 h by Western blot, and dose–response analysis was performed (Fig. S4A-B), which showed that 30 µg/mL GSC-derived exosomes induced the highest level of SIRPA expression in macrophages. Therefore, a concentration of 30 µg/mL GSC-derived exosomes was applied in the subsequent experiments.

**Fig. 3:**
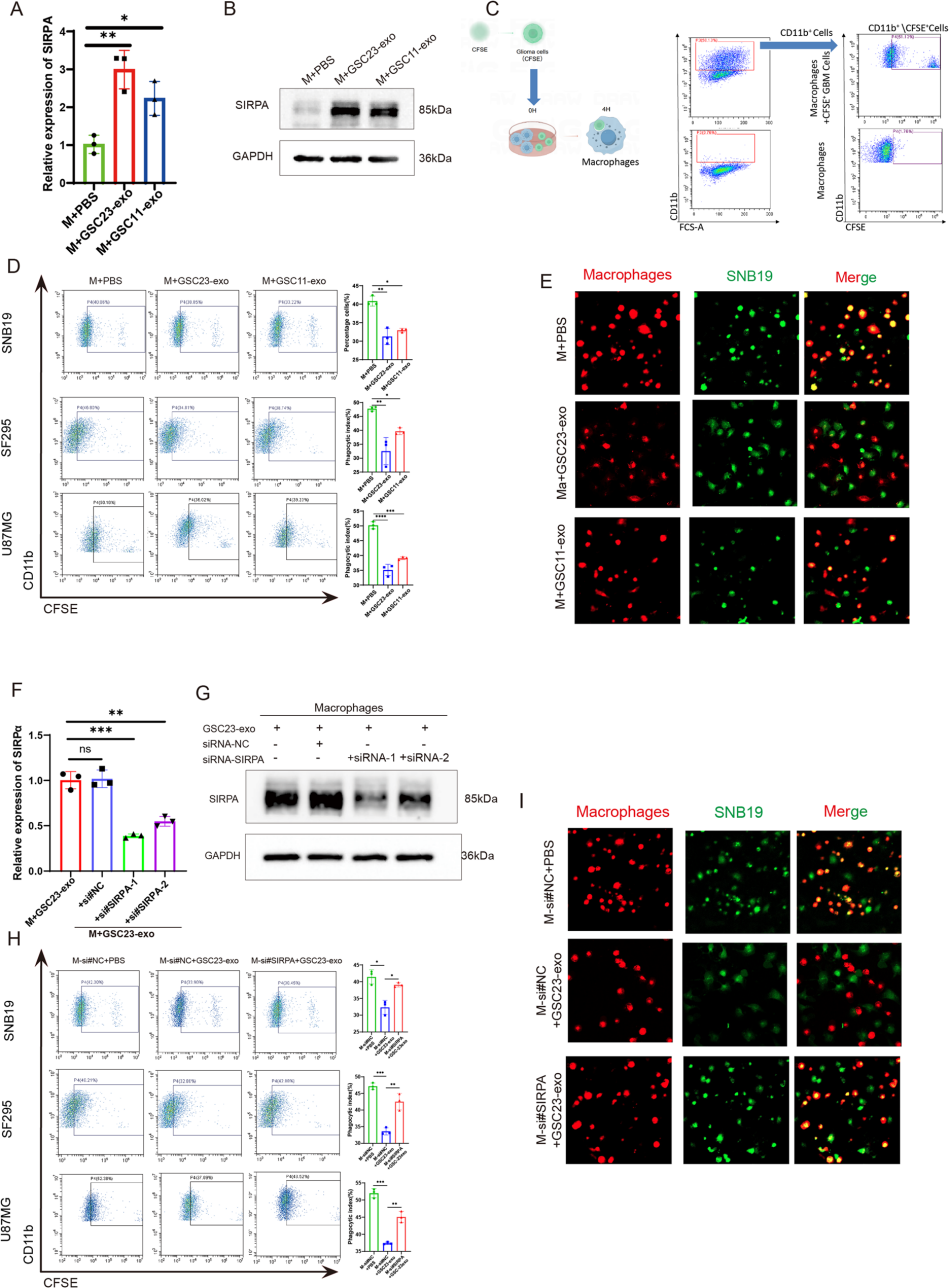
GSCs-exos suppressed phagocytosis of macrophages through upregulating SIRPA. **A**-**B** Western blot and qPCR analyses disclosed that GSCs-exos upregulated SIRPA expression of macrophages. **C** Schematic illustration of flow cytometry to evaluate phagocytic efficiency of macrophage. **D**-**E** Flow cytometry and fluorescence imaging analyses demonstrated that addition of GSCs-exos reduced the phagocytic efficiency of macrophages. **F**-**G** Western blot and qPCR analyses verified knockdown efficiency of SIRPA in macrophages. **H**-**I** Flow cytometry and fluorescence imaging disclosed that SIRPA knockdown enhanced phagocytic capacity of macrophages

The inhibitory effect of GW4869 was reversed upon supplementation with exogenous GSCs-exos (Fig. 2N–O). Western blot and qPCR analyses demonstrated that, compared with exosomes derived from either NHAs or glioblastoma cells (SNB19 and SF295), only GSC-derived exosomes significantly induced SIRPA upregulation in macrophages (Fig. S5A–B). Furthermore, GSC-derived exosomes induced SIRPA expression in macrophages independently of macrophage CD47 expression (Fig. S6A–C).

To explore the differences in molecular characteristics between GSCs and non-GSC tumor cells, GSC-like cells were defined as tumor cells expressing CD133, CD15, or OCT4 in combination with SOX2 expression, in accord with previous studies [18] (Fig. S7A). Bioinformatic analysis was performed to compare the relevant transcriptomic and molecular functional differences between GSCs and non-GSCs tumor cells (Fig. S7B-C). Enrichment analysis disclosed that GSCs were enriched in pathways and functions associated with hypoxia, epithelial-mesenchymal transition, compared with those of non-GSC-like tumor cells.

### GSC-derived exosomes suppressed macrophage phagocytic activity by upregulating SIRPA

Addition of GSCs-exos (30 µg/mL) for 2 days promoted SIRPA expression of macrophages (Fig. 3A). Then phagocytic efficiency of macrophages against glioblastoma cells was investigated with flow cytometry–based assay (Fig. 3B), which revealed that addition of GSCs-exos (30 µg/mL) for 2 days suppressed the phagocytic activity of macrophages against glioblastoma cells (Fig. 3D–E). Down-regulation of SIRPA partially reversed the suppressive effect of GSCs-exos on phagocytosis of macrophages against glioblastoma cells (Fig. 3F–I). In addition, bioinformatic analysis disclosed that expression of CD163, CD206, and HMOX1 differed significantly between SIRPA⁺ TAMs and SIRPA⁻ TAMs, whereas no obvious differences were observed in expression of TREM2, APOE, and MARCO between SIRPA⁺ TAMs and SIRPA⁻ TAMs (Fig. S8A–F). Furthermore, SIRPA knockdown reduced expression of CD163, CD206, and HMOX1 of SIRPA⁺ macrophages (Fig. S8G).

### SIRPA⁺ TAMs promoted proliferation, migration, invasion, and EMT of glioblastoma cells in vitro

**Fig. 4:**
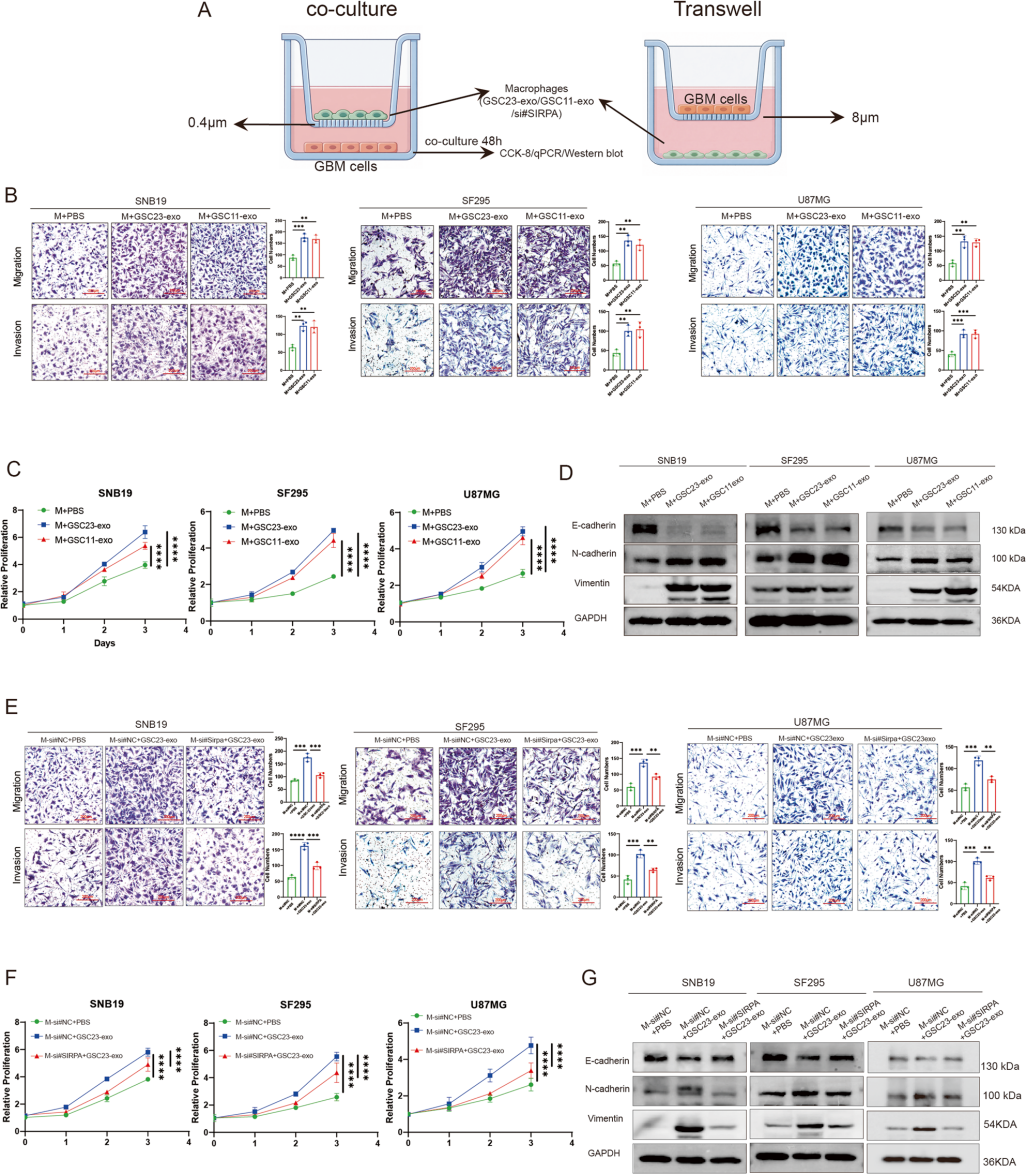
SIRPA⁺ TAMs promoted proliferation, migration, invasion, and EMT of glioblastoma cell in vitro. **A** Schematic illustration of coculture system of glioblastoma cells with macrophages. **B**-**C** Transwell and CCK-8 assay evaluating proliferation, migration, and invasion of glioblastoma cells after coculture with SIRPA⁺ TAMs. **D** Western blot analysis on EMT markers of glioblastoma cells following coculture with SIRPA⁺ TAMs. **E** Transwell and CCK-8 assay displayed that SIRPA knockdown of SIRPA⁺ TAMs decreased proliferation, migration, and invasion of glioblastoma cell. **F** Western blot analysis showing that SIRPA knockdown reversed the pro-EMT effect on glioblastoma cells induced by coculture with SIRPA⁺ TAMs

Bioinformatic analysis indicated that SIRPA⁺ TAMs possessed enhanced EMT-regulatory capacity (Fig. 1E). After induction of GSCs-exos, the functional impacts of SIRPA⁺ TAMs on glioblastoma progression were explored with co-culture system of macrophages and glioblastoma cells (Fig. 4A), which disclosed that SIRPA⁺ TAMs significantly enhanced proliferation, migration, and invasion of glioblastoma cells (Fig. 4B–C), as well as increased expression of EMT-associated proteins Vimentin and N-cadherin with decreased expression of E-cadherin in glioblastoma cells (Fig. 4D). SIRPA knockdown of TAMs partially reversed EMT-promoting effects of SIRPA⁺ TAMs on glioblastoma cells (Fig. 4E–G).

### SIRPA^+^ TAMs promoted glioblastoma progression in vivo

**Fig. 5:**
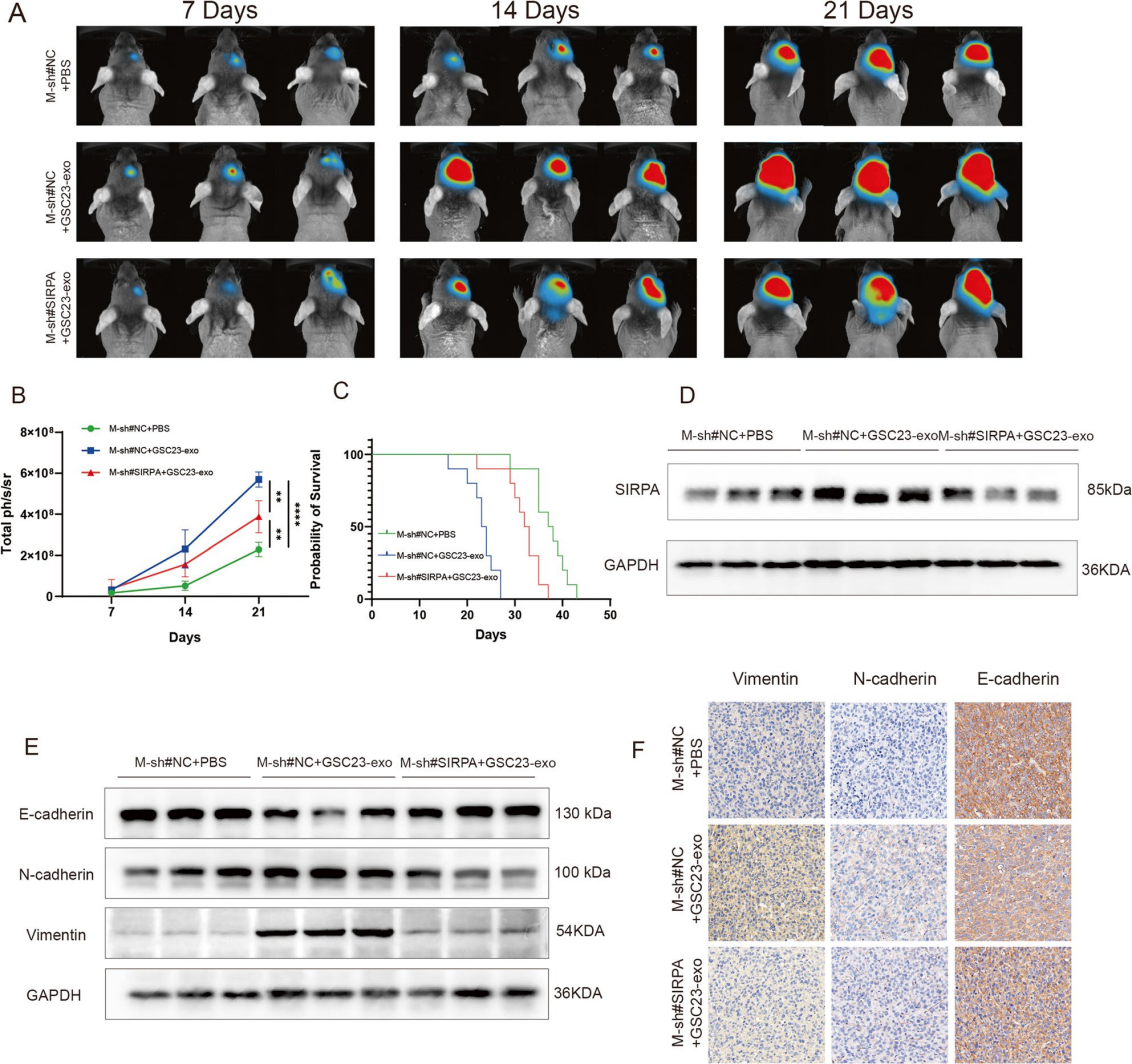
SIRPA^+^ TAMs promoted glioblastoma progression in vivo. **A**-**B** Representative bioluminescence images of intracerebral glioblastoma xenografts generated by co-implanting U87MG cells with macrophages pretreated with PBS or GSC23-exos and subsequently transfected with sh-NC or sh-SIRPA. **C** Kaplan–Meier survival analysis on mice bearing intracerebral glioblastoma. **D** Western blot analysis on SIRPA expression of intracerebral xenografts after inoculation of U87MG cells and macrophages. **E** Expression level of EMT markers in intracerebral xenografts were detected by Western blot and IHC

THP-1-derived macrophages transfected with sh-SIRPA or sh-NC were pretreated with either GSC23-derived exosomes (GSC23-exo) or PBS, and then co-implanted with 5 × 10⁵ U87MG-Luci cells at a tumor cell-to-macrophage ratio of 5:1 into the right caudate nucleus of BALB/c nude mice. Bioluminescence imaging showed that macrophages induced by GSC23-exos significantly promoted in vivo development of glioblastoma, whereas SIRPA knockdown in macrophages partially reversed the tumor-promoting effects of GSC23-exos (Fig. 5A–B). Consistently, Kaplan–Meier survival analysis demonstrated that tumor-bearing mice in GSC23-exos group exhibited poorer survival, while knockdown of SIRPA partially improved survival of tumor-bearing mice and rescued the effect of GSC23-exos in vivo (Fig. 5C). Protein expression of SIRPA increased in xenografts with macrophages induced by GSC23-exos, whereas SIRPA expression was relatively reduced with sh-SIRPA macrophages by GSC23-exos induction (Fig. 5D), which implied that macrophages induced by GSC23-exos promoted EMT of glioblastoma cells in vivo, as indicated by increased Vimentin and N-cadherin and decreased E-cadherin, whereas SIRPA knockdown partially reversed EMT of tumor cells (Fig. 5E–F).

### SIRPA+ TAMs promoted proliferation, migration, invasion, and EMT of glioblastoma cells by regulating CXCL8 in vitro

**Fig. 6:**
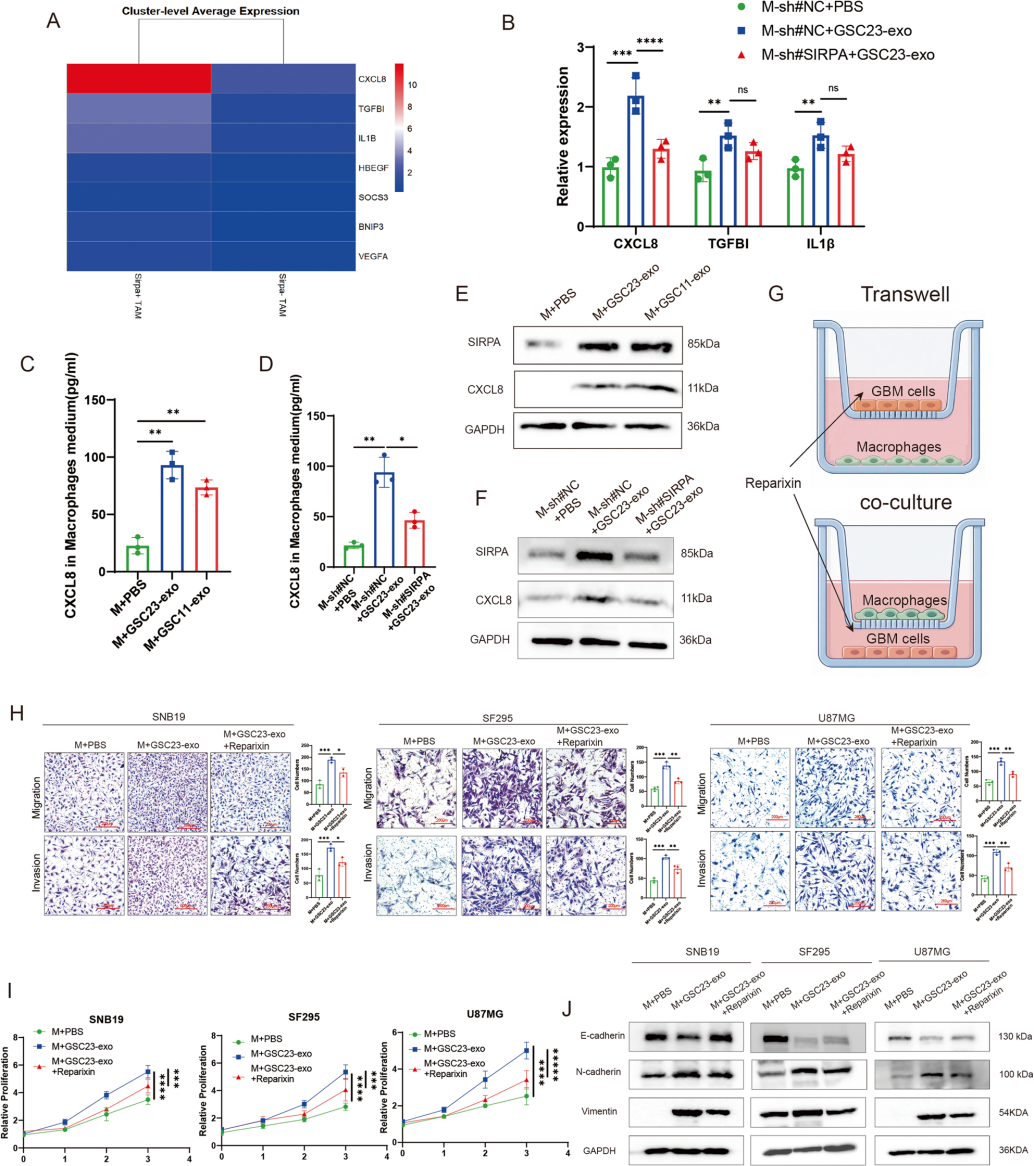
SIRPA^+^ TAMs promoted proliferation, migration, invasion, and EMT of glioblastoma cells through regulating CXCL8 secretion in vitro. **A** Comparison of cytokine expression profiles between SIRPA⁺ and SIRPA⁻ TAMs derived from 16 glioblastoma single-cell RNA-seq datasets. **B** Expression of CXCL8, TGFBI, and IL1B in macrophages with SIRPA knockdown and addition of GSC-exos. **C**-**F** Expression and secretion of CXCL8 in macrophages with SIRPA knockdown and addition of GSC-exos, determined by ELISA and Western blot. **G** Schematic illustration showing inhibition of CXCL8 signaling by addition of reparixin in macrophage–glioblastoma cells coculture system. **H**-**I** Transwell and CCK-8 assay showed that reparixin reversed the coculture-induced enhancement proliferation, migration, and invasion of glioblastoma cell mediated by SIRPA⁺ TAMs. **J** Western blot analysis of EMT markers disclosed that addition of reparixin reversed the pro-EMT effect induced by coculture of glioblastoma cells with SIRPA⁺ TAMs

Preliminary bioinformatic analysis suggested that SIRPA⁺ TAMs possessed enhanced cytokine-producing capacity, indicating the possibility that SIRPA⁺ TAMs promoted EMT of glioblastoma via regulating cytokine secretion. Therefore, cytokine expression profiles between SIRPA⁺ and SIRPA⁻ TAMs in single-cell datasets were explored, which disclosed that CXCL8, TGFBI, and IL1B as the most differentially expressed cytokines in SIRPA⁺ TAMs (Fig. 6A). Subsequent validation confirmed the positive association of the above-mentioned cytokines with SIRPA⁺ TAMs, and CXCL8 behaved the strongest correlation. ELISA and Western blot analyses disclosed that GSCs-exos induced expression and secretion of CXCL8 in macrophages, whereas SIRPA knockdown attenuated GSC-exos induced CXCL8 upregulation of macrophages (Fig. 6C–F). To investigate whether SIRPA⁺ TAMs promoted glioblastoma progression through CXCL8 secretion, CXCL8 receptor antagonist reparixin (30µM) was added to the macrophage–glioblastoma co-culture system (Fig. 6G), Transwell and CCK-8 assays revealed that reparixin inhibited proliferation, migration, and invasion effects of SIRPA⁺ TAMs on glioblastoma cells (Fig. 6H-I). Besides, reparixin reversed EMT-promoting effect on glioblastoma cells induced by SIRPA⁺ TAMs (Fig. 6J).

### GSCs-exos promoted polarization of SIRPA^+^ TAMs through regulating STAT3 signaling

**Fig. 7:**
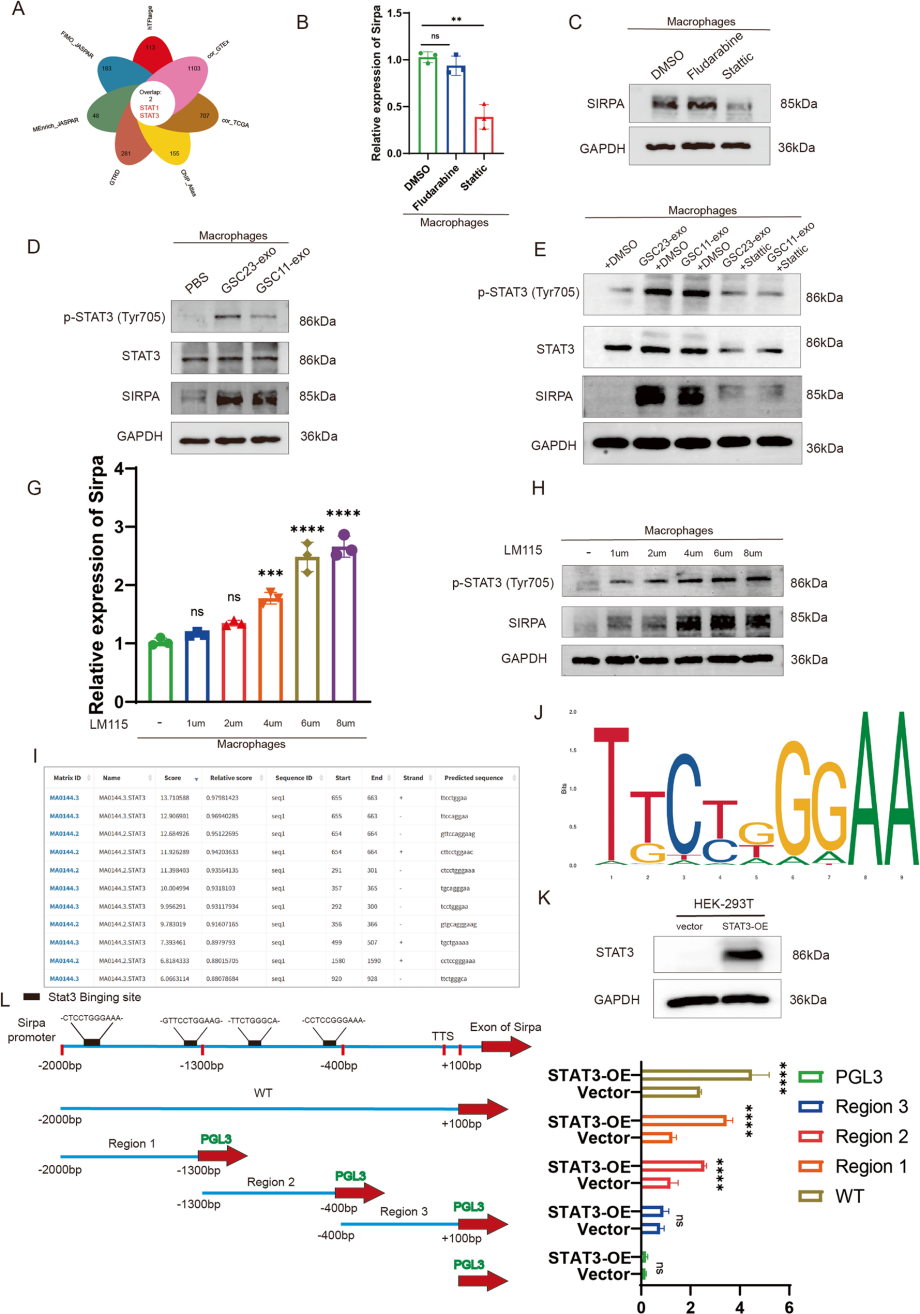
GSCs-exos promoted polarization of SIRPA^+^ TAMs through regulating STAT3 signaling. **A** Bioinformatic prediction of upstream transcription factors regulating SIRPA. **B**-**C** Effects of STAT1 and STAT3 inhibitors on SIRPA expression in macrophages determined by Western blot and qPCR. **D**-**E** Western blot analysis on expression of SIRPA, STAT3, and p-STAT3 in macrophages to evaluate the effects of GSC-exos and the STAT3 inhibitor stattic. **G**-**H** Effects of STAT3 agonist LM115 on SIRPA expression in macrophages determined by Western blot and qPCR. **I**-**J** Prediction of potential STAT3-binding sites within SIRPA promoter region and schematic representation of the binding motifs based on JASPAR database. **K** Western blot to confirm STAT3 overexpression efficiency in HEK-293T cells. **L** Verification of STAT3 binding to the SIRPA promoter and assessment of STAT3-mediated transcriptional activity with dual-luciferase reporter assay

The mechanisms by which GSCs-exos induced SIRPA⁺ TAMs had not yet been fully elucidated. On the basis of disclosing upregulation effect of GSCs-exos on both transcriptional and translational level of SIRPA of macrophages, modulating SIRPA transcription the upstream transcription factors after GSCs-exos induction were further investigated. Bioinformatic prediction was performed with JASPAR, hTFtarget, GTRD, ChIP-Atlas, GTEx, and TCGA databases, and STAT1 and STAT3 were identified as the potential transcriptional regulators of SIRPA (Fig. 7A). Then western blot and qPCR analyses were carried out, which demonstrated that the STAT3 inhibitor stattic suppressed mRNA and protein expression level of SIRPA induced by GSCs-exos, whereas the STAT1 inhibitor fludarabine had minimal effect on SIRPA (Fig. 7B–C). Besides, GSCs-exos promoted STAT3 phosphorylation and SIRPA expression, while stattic attenuated these effects. Western blot and qPCR analyses further confirmed that LM115 (a STAT3 agonist) increased mRNA and protein expression of SIRPA (Fig. 7G–H). The potential STAT3-binding sites were predicted by JASPAR database to further validate whether STAT3 directly bound to the SIRPA promoter to enhance its transcription, and the schematic binding motifs were generated (Fig. 7I-J). Based on these sites, the SIRPA promoter region was truncated into three fragments, and STAT3 overexpression efficiency was confirmed in HEK293T cells by Western blotting (Fig. 7K). Dual-luciferase reporter assay demonstrated that STAT3 binding to the SIRPA promoter significantly enhanced transcriptional activity, with Region 1 (-2000 bp to -1300 bp) exhibiting the strongest binding activity, Region 2 (-1300 bp to -400 bp) showing moderate activity, and Region 3 (-400 bp to + 200 bp) displaying minimal activity (Fig. 7L).

### GSC-derived exosomes promoted STAT3 activation through exosomal lncRNA NEAT1 carrying

**Fig. 8:**
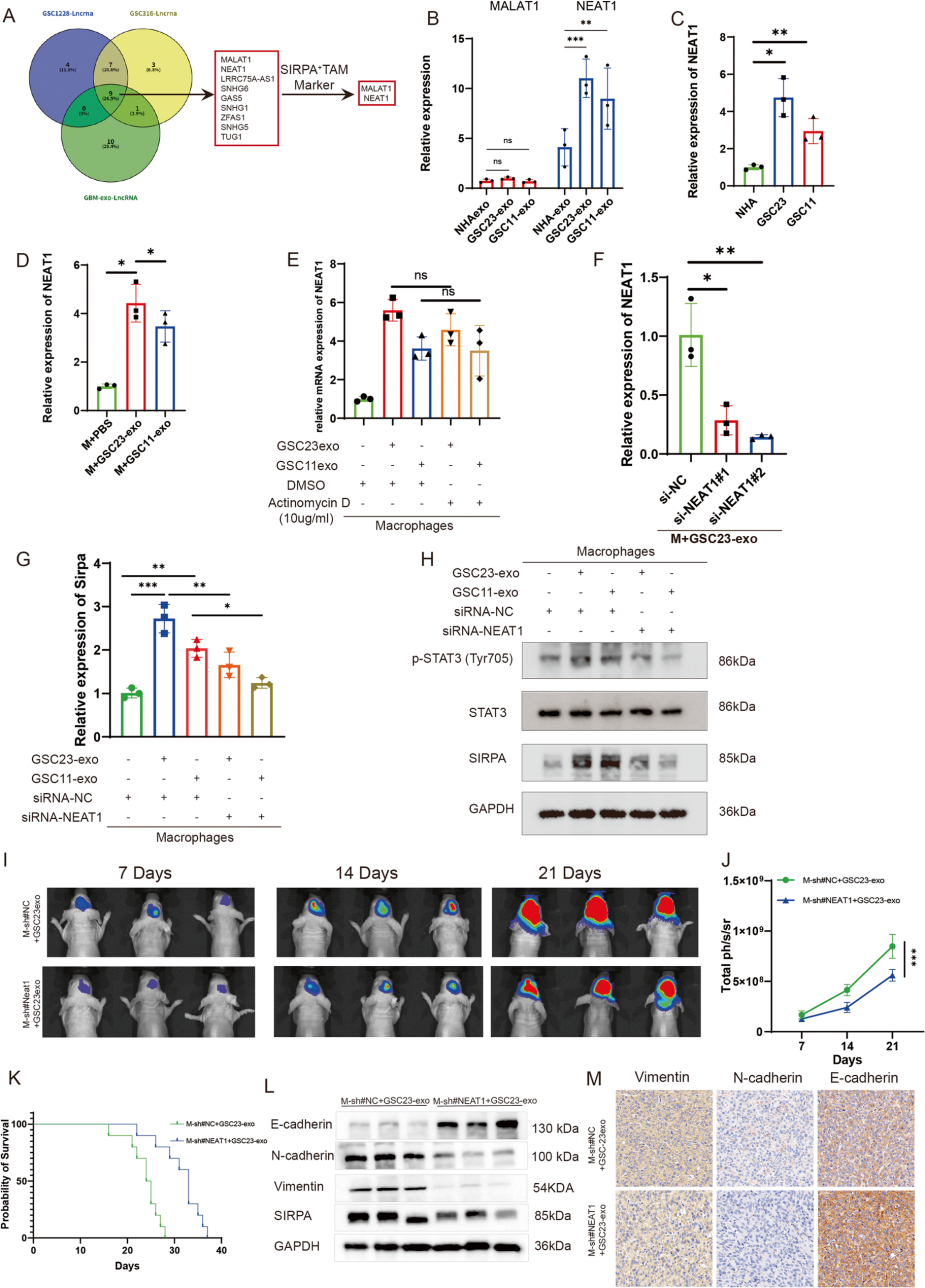
GSCs-exos promoted STAT3 activation by NEAT1 carrying. **A** Bioinformatic analysis identified potential lncRNAs from GSCs that could induce SIRPA expression. **B**-**C** qPCR analysis on MALAT1 and NEAT1 expression of GSC23, GSC11 cells, and the corresponding exosomes. **D**-**E** NEAT1 expression in macrophages stimulated with GSCs-exos determined by qPCR. **F** Knockdown efficiency of NEAT1 in macrophages determined by qPCR. **G**-**H** Effects of NEAT1 knockdown on SIRPA, STAT3 and p-STAT3 expression determined by Western blot. **I**-**J** Bioluminescence imaging on growth of intracerebral xenografts by intracranial inoculation of U87MG cells and sh-NC or sh-NEAT1 macrophages stimulated with GSC23-exos. **K** Kaplan–Meier survival analysis on tumor-bearing mice. **L**-**M** Expression of SIRPA and EMT markers in intracerebral tumors detected by Western blot and IHC

Increasing evidence has indicated that lncRNAs were selectively enriched and remained highly stable within exosomes, enabling them to serve as crucial mediators of intercellular communication. Our previous findings revealed that GSCs-exos induced SIRPA⁺ TAMs primarily through STAT3 phosphorylation, rather than altering mRNA stability or ribosomal activity of STAT3, suggest the possibility exosomal lncRNAs of GSCs might activate STAT3 phosphorylation. Bioinformatic analyses were performed to identify lncRNAs highly expressed in GSCs, glioblastoma-derived exosomes, and SIRPA⁺ TAMs, among which lncRNA MALAT1 and NEAT1 were the most prominent candidates (Fig. 8A). Quantitative PCR analysis revealed that NEAT1 was markedly enriched in GSC23 and GSC11 cells, as well as in their corresponding exosomes (Fig. 8B–C). qPCR analyses revealed that NEAT1 was predominantly enriched in GSC-derived exosomes, whereas no significant enrichment was observed in exosomes derived from NHAs or glioblastoma cell lines (Fig. S5C). Co-culture of macrophages with GSCs-exos significantly increased intracellular level of NEAT1, and this effect was not reversed by transcriptional inhibitor Actinomycin D (Fig. 8D–E). Knockdown of NEAT1 in SIRPA⁺ TAMs suppressed the transcriptional and translational levels of SIRPA and inhibited STAT3 phosphorylation as well (Fig. 8F–H).

Macrophages transfected with sh-NEAT1 or sh-NC were pretreated with GSC23-exo (30 µg/mL) for 2 days and then co-implanted with 5 × 10⁵ U87MG-Luci cells at a tumor cell-to-macrophage ratio of 5:1 into the right caudate nucleus of BALB/c nude mice to observe the in vivo effect of macrophages with NEAT1 knockdown on tumor development. Bioluminescence imaging revealed that NEAT1 knocking down in macrophages partially reversed the tumor-promoting effects of GSC23-exo activated macrophages (Fig. 8I–J). Consistently, Kaplan–Meier survival analysis indicated that NEAT1 silencing partially rescued the survival disadvantage induced by GSC23-exo in vivo (Fig. 8K). NEAT1 knockdown partially reversed the promoting effects of GSC23-exos on SIRPA upregulation of macrophages and the alterations in EMT markers of glioblastoma in xenografts tissues (Fig. 8L–M).

To determine the effect of lncRNA NEAT1 on inducing SIRPA⁺ macrophages through upregulation of the HSP90B1-STAT3 axis, overexpression of NEAT1 in macrophages was achieved through plasmid vector transfection, and verified the overexpression efficiency by qPCR assay (Fig. S9A). Western blot results showed that NEAT1 overexpression markedly increased the expression of SIRPA, p-STAT3, and HSP90B1 (Fig. S9B). Furthermore, exposure to Luminespib (HSP90B1 inhibitor) following NEAT1 overexpression suppressed SIRPA expression and STAT3 phosphorylation, whereas subsequent exposure to LM115 (STAT3 activator) restored SIRPA expression (Fig. S9C). These results suggested that NEAT1 induced SIRPA expression in macrophages, which mainly depended on the HSP90B1-STAT3 axis.

PBMCs were isolated from whole blood samples of GBM patients and induced into MDMs (Fig. S10A), as verified with flow cytometry (Fig. S10B). Western blot assay demonstrated that GSC-derived exosomes induced prominent conversion of MDMs into SIRPA⁺ macrophages, whereas exosomes from NHAs, GBM cells (SNB19 and SF295), as well as PBS, did not show obvious effects (Fig. S10C). Furthermore, GSC-derived exosomes upregulated the expression of SIRPA, p-STAT3 and HSP90B1 in MDMs (Fig. S10D). Upregulation of SIRPA and p-STAT3 in MDMs was dependent on HSP90B1 (Fig. S10E). Additionally, Western blot revealed that GSC-derived exosomal NEAT1 upregulated the expression of SIRPA, p-STAT3 and HSP90B1 as well (Fig. S10F), which implied that GSC-derived exosomal NEAT1 induced the conversion of MDMs into SIRPA⁺ macrophages by upregulating the HSP90B1-STAT3 axis.

To further clarify whether in situ tumor development was driven by exosomal NEAT1 of GSCs, intracranial xenografts were established by co-injecting 5 × 10⁵ U87MG GBM cells and 1 × 10⁵ macrophages together with either 30 µg shNC-GSC23 exosomes, or 30 µg shNEAT1-GSC23 exosomes, or PBS. Dynamic fluorescence tracing on tumor-bearing mice showed that shNC-GSC23 exosomes significantly promoted intracranial tumor progression and shortened survival of tumor-bearing mice, compared with shNEAT1-GSC23 exosomes group (*P* < 0.001) (Fig. S11A–C).

### NEAT1 promoted STAT3 activation by modulating HSP90B1 stability

**Fig. 9:**
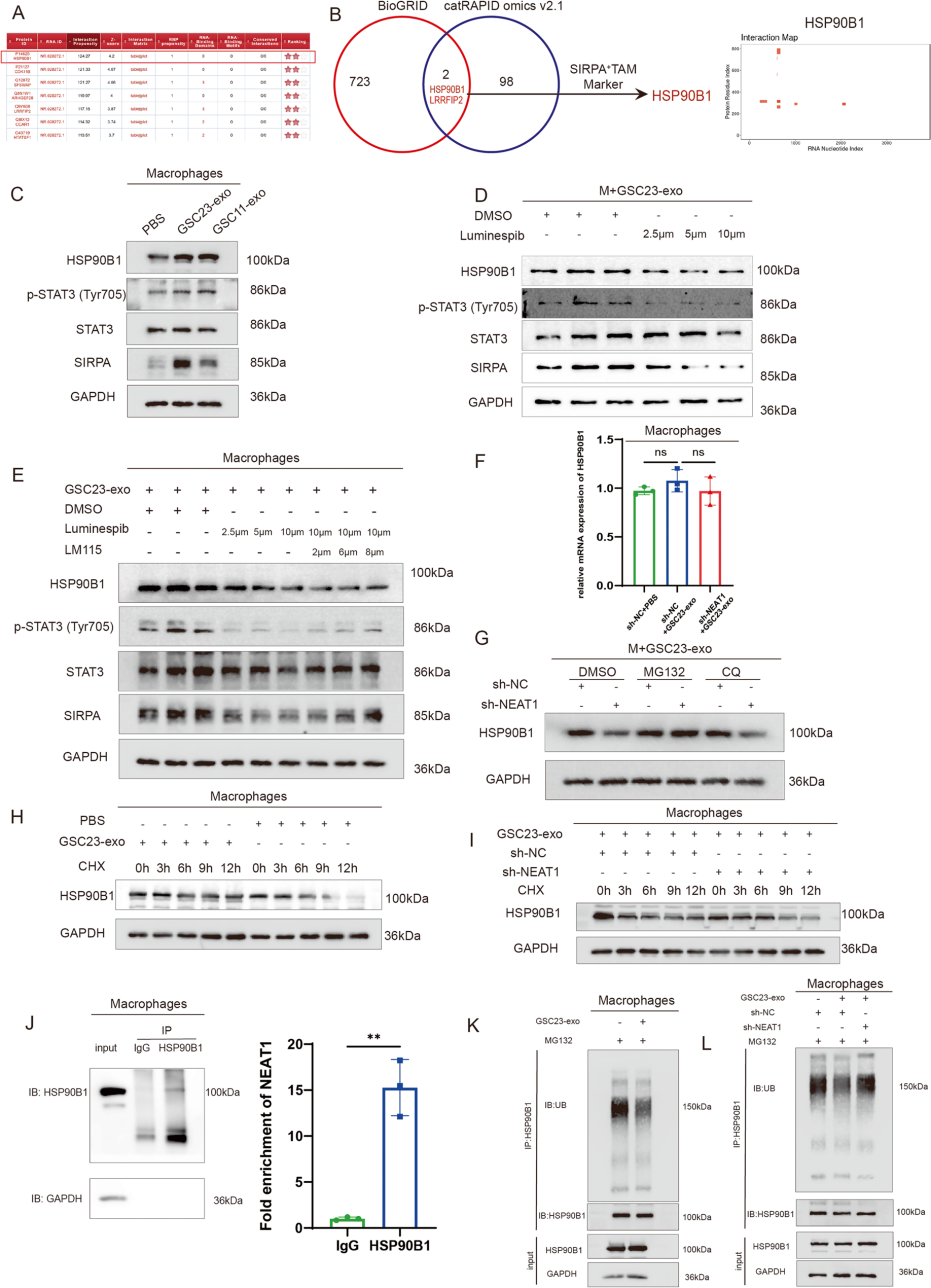
NEAT1 promoted STAT3 activation by modulating HSP90B1 stability. **A** Prediction of NEAT1-binding proteins and their potential interaction sites with catRAPID omics analysis. **B** Bioinformatic analysis identifying proteins that potentially interact with both STAT3 and NEAT1, which were differentially expressed in SIRPA⁺ TAMs. **C** HSP90B1 expression in macrophages stimulated with GSCs-exos detected by Western blot. **D**-**E** Western blot analysis showing that STAT3 phosphorylation induced by GSC23-exos was dependent on HSP90B1. **F** qPCR assay showed that neither GSC23-exos nor NEAT1 knockdown altered mRNA level of HSP90B1. **G** Western blot analysis showing that NEAT1 contained in GSC23-exos upregulated HSP90B1 expression through the proteasomal pathway. **H**-**I** Western blot analysis was performed to verify that NEAT1 contained in GSC23-exos upregulated HSP90B1 expression by inhibiting its proteasome-mediated degradation. **J** RIP assay confirmed that NEAT1 physically interacted with HSP90B1 in macrophages. **K**-**L** Co-IP assay showing that GSC23-derived exosomal NEAT1 reduced HSP90B1 ubiquitination

Bioinformatic prediction with catRAPID omics were performed to screen proteins that might directly bind to NEAT1, which disclosed that NEAT1 did not directly interact with STAT3 (Fig. 9A), indicating that NEAT1 might bind to other proteins that activate STAT3 phosphorylation. Subsequently, proteins that directly interact with STAT3 were predicted with BioGRID database to screen out to bind both NEAT1 and STAT3, as well as high expression in SIRPA⁺ TAMs. Among these candidates, HSP90B1 was identified as the most suitable target to serve as the potential mediator (Fig. 9B). Western blot analysis revealed that GSCs-exos promoted HSP90B1 protein expression in macrophages (Fig. 9C). Treatment with a novel HSP90B1 inhibitor for 2 days partially reversed the GSCs-exos–induced upregulation of STAT3 phosphorylation and SIRPA expression of macrophages, whereas addition of LM115 partially restored the upregulation effects (Fig. 9D–E). Besides, GSC23-exos plus NEAT1 knockdown did not alter HSP90B1 mRNA level of HSP90B1, indicating that NEAT1 regulated HSP90B1 primarily at the post-transcriptional level (Fig. 9F). Furthermore, Western blot analysis showed that the proteasome inhibitor MG132, partially restored HSP90B1 expression suppressed by NEAT1 knockdown, but neither DMSO nor the lysosomal inhibitor chloroquine (CQ) (Fig. 9G), suggesting that NEAT1 can enhance functional status of HSP90B1 by inhibiting its ubiquitination. Consistently, GSC23-exos prolonged protein half-life of HSP90B1, whereas NEAT1 knockdown partially reversed this effect (Fig. 9H-I). RIP assay confirmed that HSP90B1 immunoprecipitates were enriched for NEAT1, indicating a direct interaction between HSP90B1 and NEAT1 (Fig. 9J). Western blotting disclosed that GSC23-exos inhibited ubiquitination of HSP90B1, whereas NEAT1 silencing partially reversed this inhibition effect (Fig. 9K-L).

### NEAT1 facilitated the interaction between HSP90B1 and STAT3

**Fig. 10:**
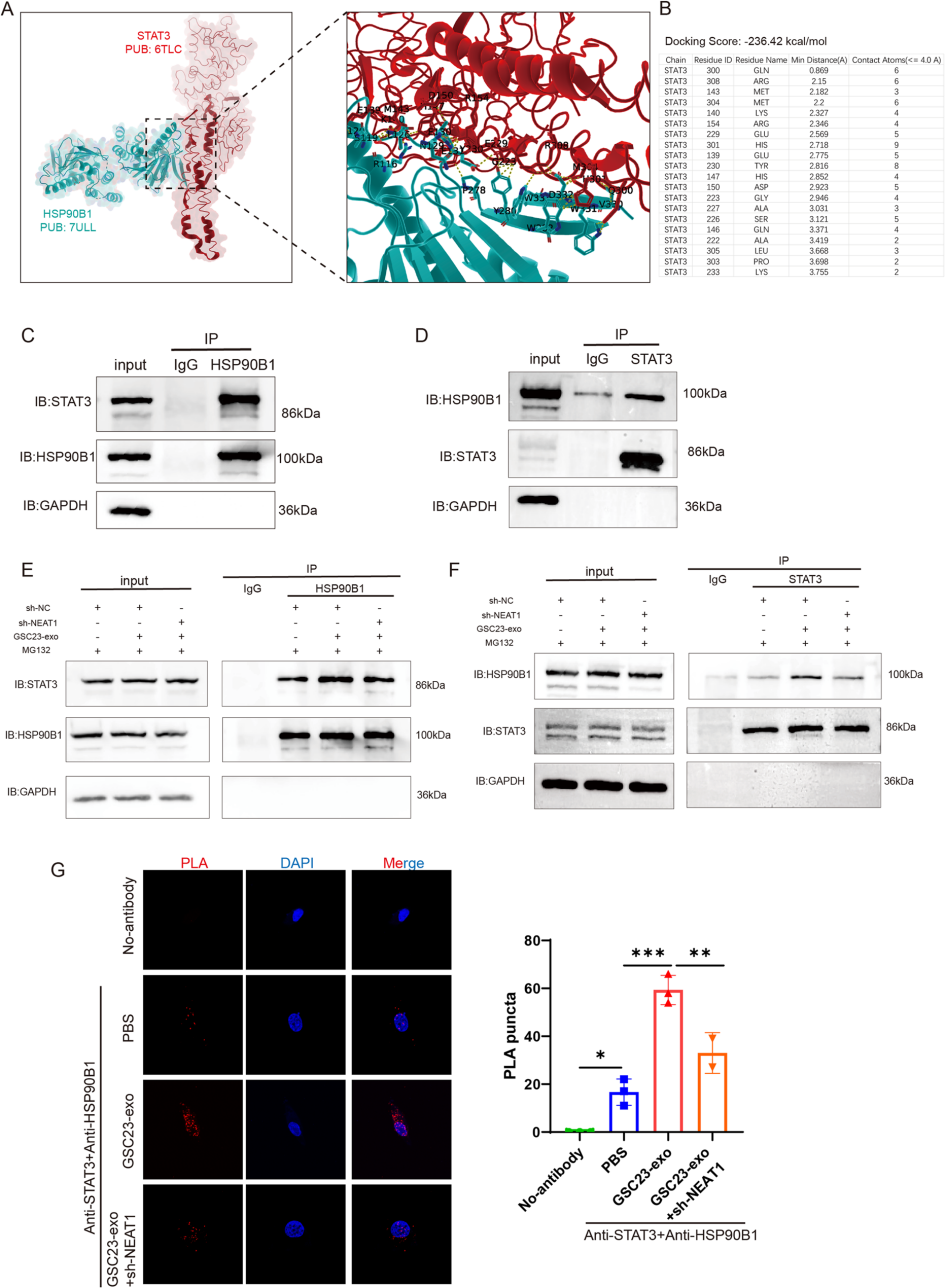
NEAT1 facilitated the interaction between HSP90B1 and STAT3. **A**-**B** Schematic representation of the molecular docking between STAT3 and HSP90B1. The protein structure of STAT3 was shown in red cartoon form, whereas HSP90B1 was depicted in blue. The binding interface was highlighted with stick representations, and hydrogen bonds were indicated by yellow dashed lines. **C**-**D** Co-IP assay confirmed the physical interaction between HSP90B1 and STAT3 in macrophages. **E**-**G** Co-IP and PLA assay showing that GSC23-exos enhanced the interactions between HSP90B1 and STAT3 in macrophages, whereas NEAT1 knockdown partially reversed this effect

Molecular docking analysis was conducted via HDOCK server to predict the potential interaction mode and binding affinity between STAT3 (PDB: 6TLC) and HSP90B1 (PDB: 7NULL), which implied that STAT3 can interact with HSP90B1 stably, exhibiting a total binding energy of − 236.42 kcal/mol (Fig. 10A–B). The direct binding was then verified by Co-IP assays, which confirmed interaction between HSP90B1 and STAT3 in macrophages (Fig. 10C–D). Addition of GSC23-exos enhanced binding between HSP90B1 and STAT3, whereas NEAT1 knockdown partially reversed this effect (Fig. 10E–G). These findings indicated that NEAT1 enhanced interactions between HSP90B1 and STAT3, thereby facilitating STAT3 phosphorylation in macrophages.

## Discussion

TME of glioblastoma was highly complex, consisting of diverse cellular and noncellular components that collectively influenced tumor progression, immune evasion, and therapeutic resistance [19]. Among these cellular constituents, TAMs represented a major and functionally versatile population that played pivotal roles in shaping highly immunosuppressive landscape of glioblastoma [6]. TAMs interacted extensively with glioblastoma cells and other immune components, thereby promoting tumor growth, invasion, angiogenesis, and therapeutic resistance [19]. Traditionally, TAMs had been classified into two major subtypes: proinflammatory M1-like TAMs and anti-inflammatory M2-like TAMs [20]. However, recent studies revealed that macrophages in nearly all cancer types exhibited co-expression of both M1-like and M2-like gene signatures within the same subpopulations [10, 21, 22], implying that the classical dichotomous model of M1/M2 TAMs was overly simplified and failed to capture the functional heterogeneity of TAMs in glioblastoma [13]. Single-cell transcriptomic technologies had emerged as powerful tools to resolve this cellular heterogeneity at high resolution, which helped to identify a distinct subpopulation of TAMs characterized by high expression of SIRPA in the current studies. The SIRPA^+^ TAMs have represented an immunosuppressive and tumor-promoting macrophage phenotype in glioblastoma. Furthermore, we elucidated the mechanisms by which GSCs-exos drove the polarization of SIRPA⁺ TAMs and promoted immune evasion and tumor progression in glioblastoma.

SIRPA was a transmembrane immunoglobulin superfamily receptor predominantly expressed on myeloid cells, where it interacted with CD47 to deliver a “don’t eat me” signal that inhibited phagocytosis [23]. Numerous preclinical studies demonstrated that blockade of the SIRPA–CD47 axis enhanced phagocytic activity of macrophages against tumor cells, and effectively suppressed tumor growth in multiple cancer models [24, 25]. However, the development of CD47-blocking therapies had faced significant challenges in recent years, mainly due to on-target hematologic toxicity and limited clinical efficacy [26, 27]. Therefore, targeting SIRPA on TAMs or its upstream regulatory pathways might have represented a more rational and effective immunotherapeutic strategy to overcoming immune evasion in glioblastoma. SIRPA⁺ TAMs constituted a crucial immunoregulatory subset within TME of glioblastoma. SIRPA knockout reprogrammed TAMs toward a status with antitumor and proinflammatory activities, characterized by enhanced phagocytic, antigen-presenting capacities, thereby improving therapeutic efficacy in preclinical models of colorectal and pancreatic cancer [28]. In a murine lung cancer model, SIRPA knockout induced TAM polarization toward an M1-like phenotype and reduced IL-6 secretion, thereby suppressing tumor progression [29]. Targeted depletion of SIRPA⁺ TAMs enhanced the expression of MHC molecules and costimulatory signals, thereby promoting CD8⁺ T-cell proliferation and recruitment. This process initiated TME reprogramming and eliminated solid tumors by restoring both innate and adaptive immune responses [30]. Previous studies on SIRPA⁺ TAMs in glioblastoma had mainly relied on bioinformatic analyses, and the cellular origin of these macrophages, as well as the corresponding signaling axis underlying their interactions with tumor cells remained largely unelucidated. Mechanistically, our studies demonstrated that NEAT1 encapsulated within GSCs-exos bound to and stabilized HSP90B1, the latter directly interacted with STAT3 and enhanced its phosphorylation-dependent transcriptional activity. Consequently, activated STAT3 transcriptionally upregulated SIRPA, leading to the induction of immunosuppressive SIRPA⁺ TAMs. Functionally, SIRPA⁺ TAMs displayed diminished phagocytic capacity against glioblastoma cells, consistent with their immunosuppressive phenotype and reduced ability to eliminate tumor cells. Intriguingly, our results revealed that SIRPA⁺ TAMs secreted high level of CXCL8, which drove epithelial–mesenchymal transition (EMT) and promoted glioblastoma progression. Notably, this protumorigenic effect occurred independently of the SIRPA–CD47 signaling axis, suggesting additional, non-phagocytic mechanisms by which SIRPA⁺ TAMs promoted glioblastoma progression. Similarly, recent studies have found that deletion of SIRPA in macrophages enhanced both innate and adaptive immune activation independent of its interaction with CD47 [30]. Our study highlights that SIRPA⁺ macrophages tended to exhibit an immunosuppressive phenotype characterized by high expression of CD163, CD206, and HMOX1. SIRPA may potentially contribute to the polarization of macrophages toward this immunosuppressive state; however, these findings still require further validation with more robust mechanistic evidence.

NEAT1 was transcribed into two isoforms from a common promoter, the short transcript NEAT1-1 (3.7 kb) and the long transcript NEAT1-2 (22.7 kb), which differed in their 3′-end processing and biological functions [31]. The long isoform NEAT1-2 was predominantly localized in cell nucleus, where it served as a core architectural scaffold of paraspeckles and was essential for their biogenesis, stability, and structural integrity [32]. Unlike NEAT1-2, NEAT1-1 was not required for the structural organization of paraspeckles [33], it modulated diverse biological processes in cancer cells by interacting with mRNAs, DNA, and multiple RNA-binding or chromatin-associated proteins [34]. NEAT1 served as a scaffold for EZH2 to regulate WNT/β-catenin pathway and promote glioblastoma progression [35]. NEAT1-1 was selectively packaged into exosomes to mediate intercellular communication. Multiple myeloma cell–exosomal NEAT1 modulated the EZH2/PBX1 signaling axis, inhibited NK-cell cytotoxicity, and facilitated immune evasion of multiple myeloma cells [36]. Exosomal NEAT1 derived from HUVECs promoted M2 polarization of macrophages through DDX3X/NLRP3 pathway, thereby facilitating osteogenesis [37]. Our studies revealed that GSCs highly expressed NEAT1 and rich in their exosomes. After engulfed by macrophages, exosomal NEAT1 promoted SIRPA⁺ polarization of macrophages, thereby facilitating immune evasion and promoting malignant progression of glioblastoma. These findings suggested that NEAT1 might represent a potential therapeutic target for reversing immunosuppression and improving immunotherapeutic efficacy against glioblastoma.

As a convergence point for multiple oncogenic signaling pathways, STAT3 was central to the regulation of antitumor immune responses [38]. STAT3 was primarily activated through ligand-induced phosphorylation at tyrosine 705, which promoted its dimerization, nuclear translocation, DNA binding, and subsequent transcriptional activity [39]. STAT3 played a critical regulatory role in maintaining the immunosuppressive phenotype of TAMs and contributed to immune evasion in glioblastoma [40]. HSP90B1 (gp96/GRP94) was a member of the heat shock protein 90 (HSP90) family [41]. As a protein molecule STAT3 was not a traditional client of HSP90 family, and did not rely on them for de novo folding or maturation, it still depended on HSP90 family members for activation, nuclear translocation, and DNA binding [42]. The current studies revealed that the selective HSP90B1 inhibitor suppressed STAT3 phosphorylation significantly, thus reduced expression of its downstream target, SIRPA. Addition of STAT3 agonist (LM115) restored SIRPA expression, indicating that STAT3 phosphorylation was dependent on HSP90B1. Besides, GSC-derived exosomal NEAT1 reduced HSP90B1 ubiquitination and degradation, thereby promoting HSP90B1-dependent STAT3 phosphorylation. Furthermore, knockdown of NEAT1 decreased direct interactions between HSP90B1 and STAT3, suggesting that NEAT1 was involved in facilitating their binding.

A limitation of this study was the use of an immunodeficient xenograft model, which restricted a comprehensive evaluation of adaptive immune interactions in polarization of SIRPA⁺ TAMs and tumor immune evasion. This limitation can be addressed by incorporating humanized immune-reconstituted animal model or organoid model, together with multi-omics analysis on clinical samples and additional functional validation.

**Fig. 11:**
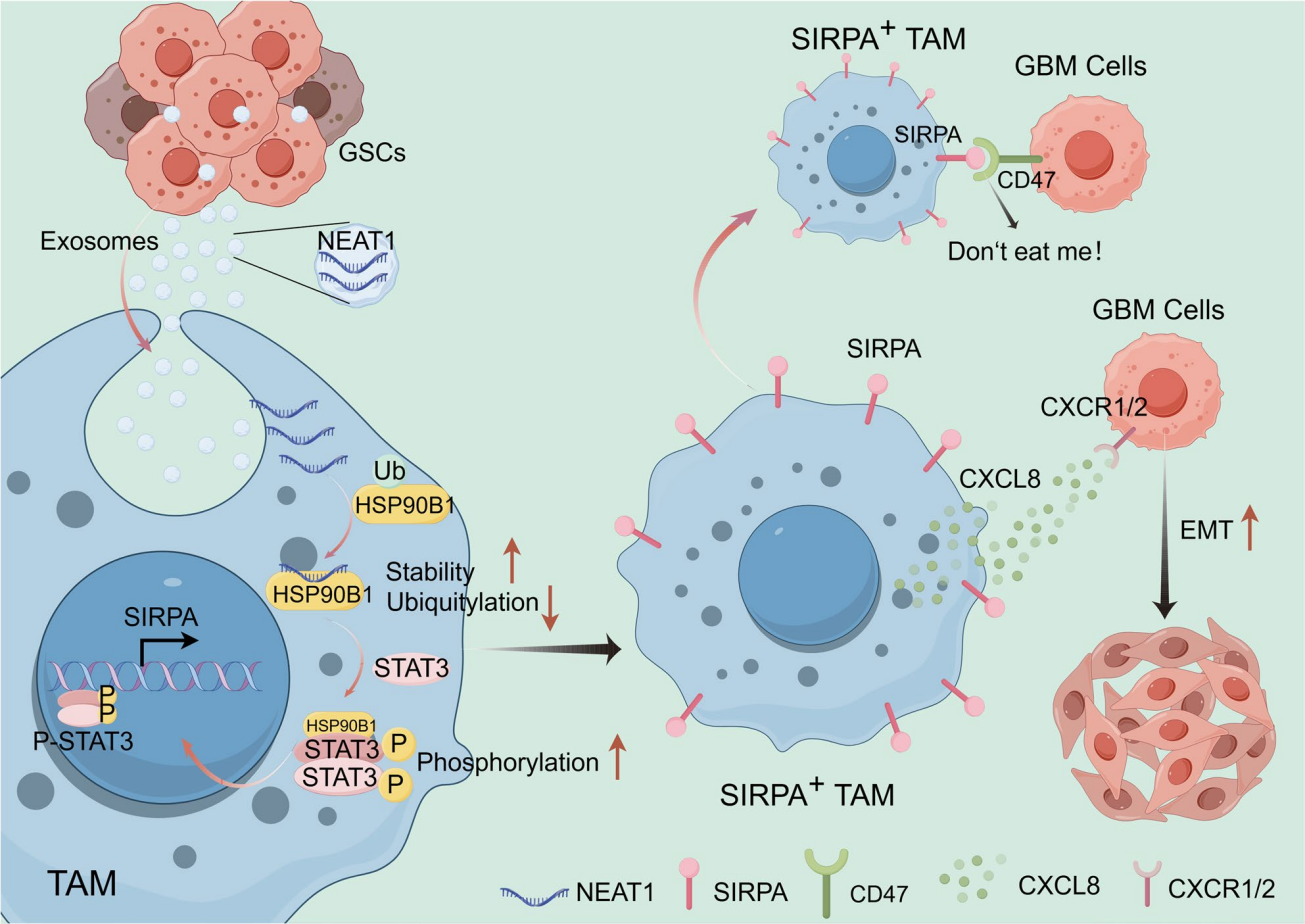
A graphical abstract for this study

In summary, our study elucidated the mechanism by which GSC-derived exosomal NEAT1 induced SIRPA⁺ TAMs through the HSP90B1/STAT3 axis, thereby promoting immune evasion and malignant progression of glioblastoma (Fig. 11). These findings highlighted that NEAT1 and SIRPA had potential to serve as therapeutic targets in disrupting immune-suppressive TME against glioblastoma. However, our study was mainly based on cellular and animal models, which differ from the complex multi-interaction of true TME of human glioblastoma to certain extent. The existence and transformation value of NEAT1/HSP90B1/STAT3/SIRPA signaling axis during GSCs initiated tissue remodeling of immunosuppressive TME in glioblastoma remains to be further verified. In addition, the interactions between SIRPA⁺ TAMs and other subpopulations of macrophages have not yet been fully clarified, which deserved further investigation.

## Conclusion

In conclusion, by integrating single-cell analysis with in vitro and in vivo experiments, the current studies highlighted that GSCs promoted immune evasion and malignant progression of glioblastoma through transferring their exosomal lncRNA NEAT1 to induce SIRPA⁺ TAMs via regulating the HSP90B1/STAT3 axis, namely, NEAT1 bound to and stabilized HSP90B1, thereby maintaining the stability of HSP90B1–STAT3 complex. These findings revealed a novel NEAT1/HSP90B1/STAT3/SIRPA signaling axis that shaped the immunosuppressive microenvironment of glioblastoma, implying that NEAT1 and SIRPA had potentials to serve as therapeutic targets for improving immunotherapy against glioblastoma.

## Supplementary Information

Supplementary Material 1.

## Data Availability

The datasets supporting the conclusions of this article are available in the GEO repository (GSE182109, https://www.ncbi.nlm.nih.gov/geo/), the TCGA repository (https://portal.gdc.cancer.gov/) and the CGGA repository (https://www.cgga.org.cn/).

## Abbreviations

NEAT1: Nuclear Paraspeckle Assembly Transcript 1
SIRPA: Signal Regulatory Protein Alpha
GSCs: Glioblastoma Stem Cells
CXCL8: C-X-C Motif Chemokine Ligand 8
HSP90B1: Heat Shock Protein 90 Beta Family Member 1
STAT3: Signal Transducer and Activator of Transcription 3
GSC-derived exosome: GSC-exo
CNS: Central Nervous System
TME: Tumor Microenvironment
TAMs: Tumor-Associated Macrophages
GBM: Glioblastoma
scRNA-seq: Single-Cell RNA Sequencing
TCGA: The Cancer Genome Atlas
GEO: Gene Expression Omnibus
HSP90: Heat Shock Protein 90
GRP94: Glucose-Regulated Protein 94
