# Supplementary material for: Exosomal NEAT1 from tumor stem cells induces SIRPA+ macrophages to enhance immune evasion and glioblastoma progression via upregulating the HSP90B1/STAT3 axis
Source: J Exp Clin Cancer Res. 2026 May 11;45:150. doi: 10.1186/s13046-026-03729-z (PMC13330394; doi:10.1186/s13046-026-03729-z)
Supplement: Supplementary file 1 — Supplementary Material 1. [file 13046_2026_3729_MOESM1_ESM.docx]

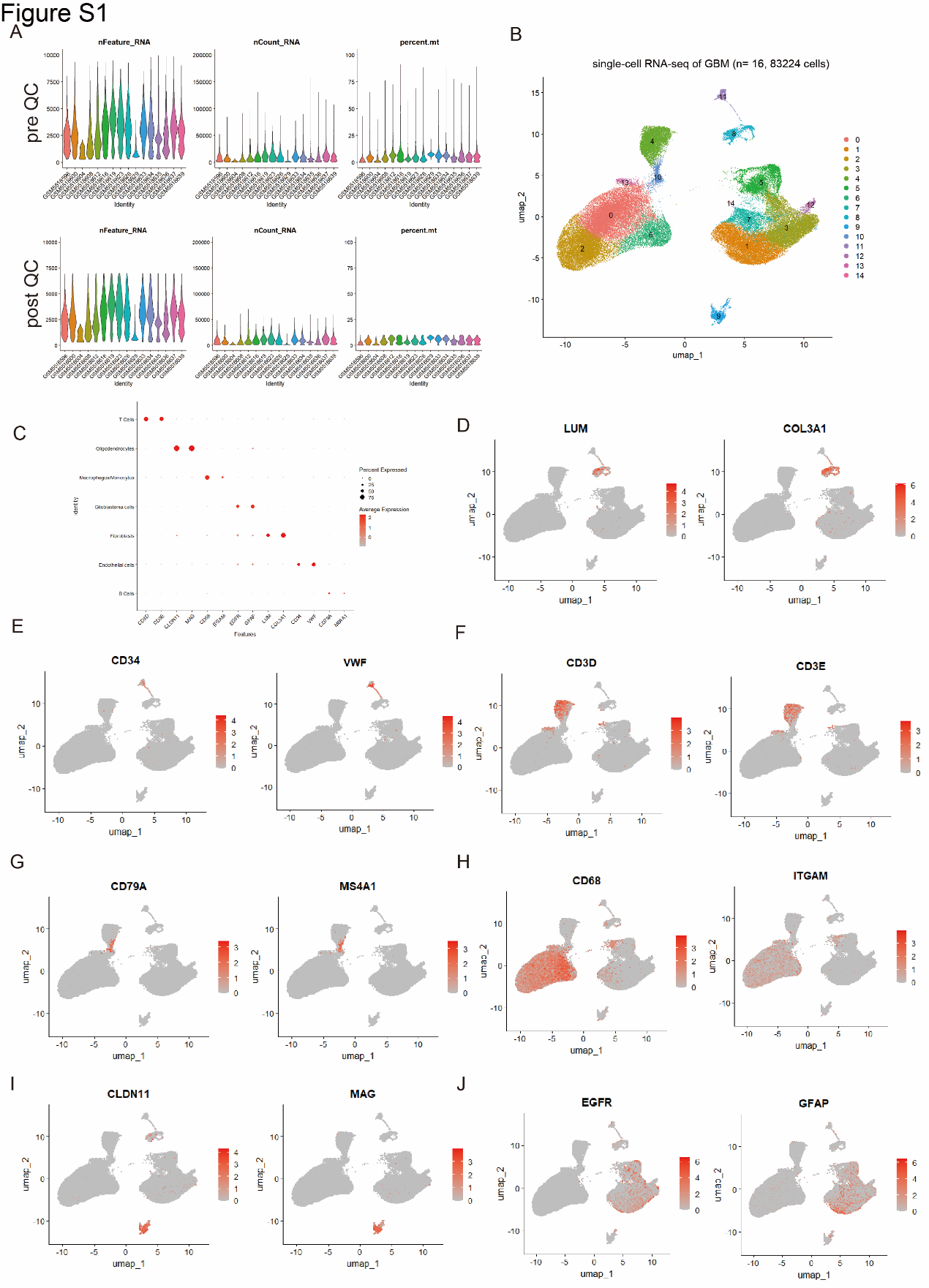


**FIG. S1. Quality control, dimensionality reduction, clustering, and annotation of single‐cell RNA-seq data.**

(A) Violin plots showing the distribution of nFeature RNA, nCount RNA, and percent.mt before and after quality control. (B) UMAP plots visualizing 15 major cell clusters in glioblastoma samples, generated after PCA-based dimensionality reduction. (C) Dot plot displaying marker genes used to define seven distinct cell clusters. (D-J) UMAP plots showing the identified markers of seven cell clusters.


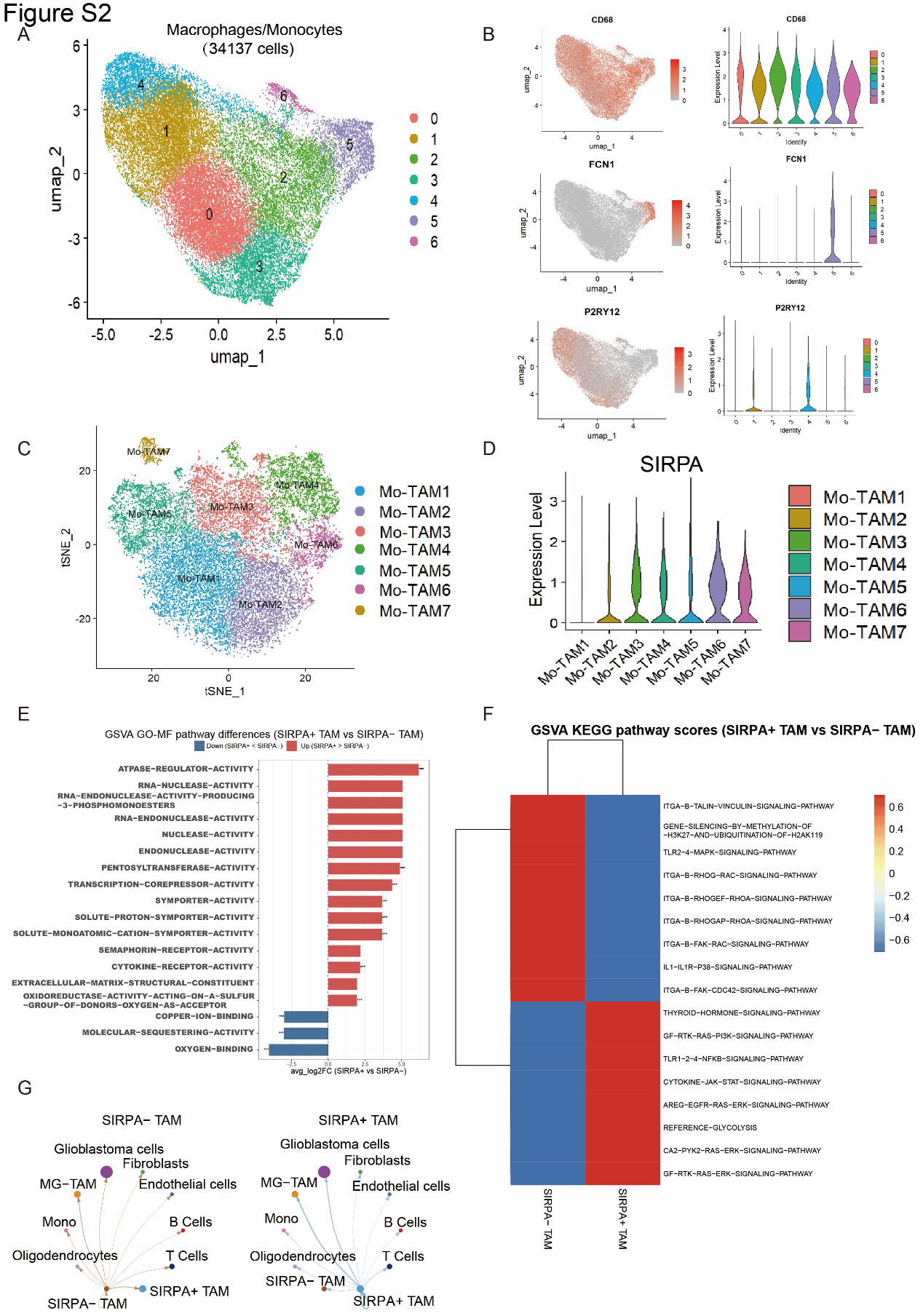


**FIG. S2. Reclustering and refined annotation of monocyte/macrophage populations.**

(A) UMAP plots visualizing 7 reclustered subpopulations of monocytes/macrophages. (B) UMAP and violin plots displaying marker genes used to identify monocyte/macrophage cell clusters. (C-D) tSNE plot showing six distinct Mo-TAM clusters and violin plot displaying differential SIRPA expression across clusters. (E-F) GSVA-based differential pathway enrichment analysis between SIRPA⁺ and SIRPA⁻ TAMs. (G) CellChat analysis depicting intercellular communications between SIRPA⁺/SIRPA⁻ TAMs and other cell populations.


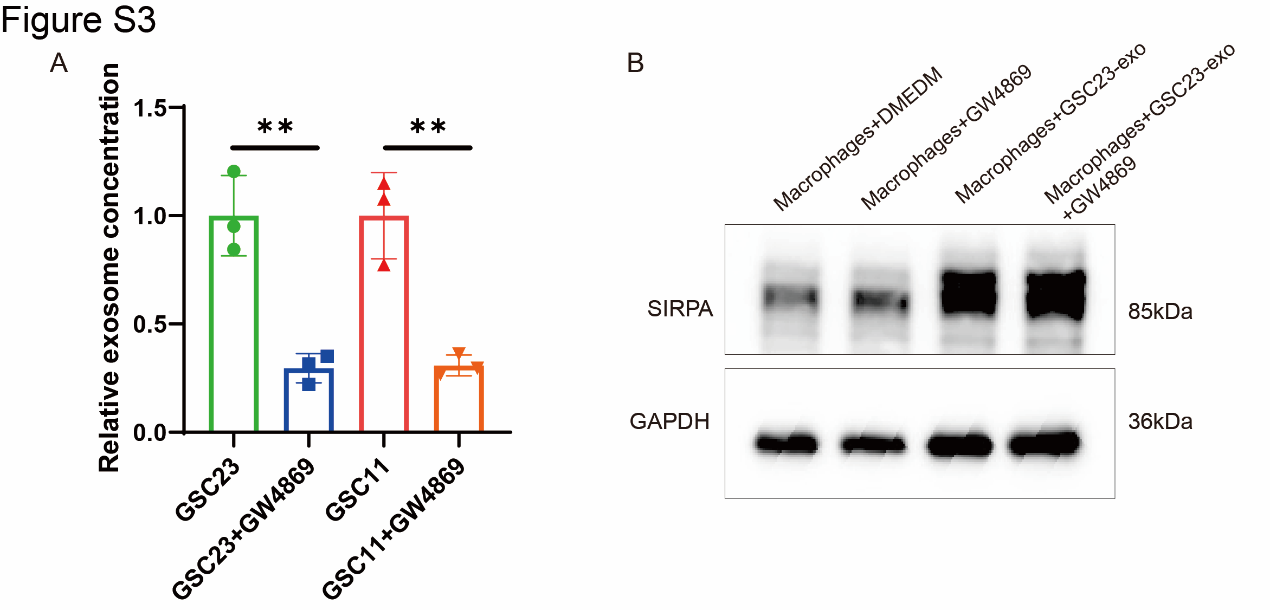


**FIG. S3. GW4869 inhibited exosome secretion from GSCs, but did not affect SIRPA expression in macrophages.** (A) Exosome secretion from GSCs decreased significantly after addition of GW4869. (B) GW4869 treatment did not affect SIRPA expression in macrophages.

**
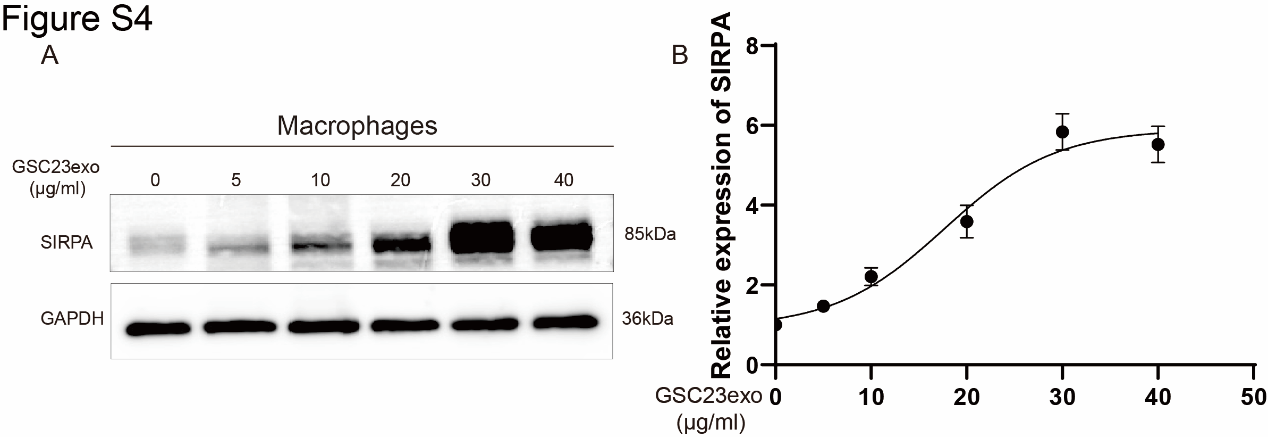
**

**FIG. S4. Effects of different concentrations of GSC-derived exosomes on SIRPA expression in macrophages.** (A) Western blot analysis of SIRPA expression in macrophages treated with 0, 5, 10, 20, 30, and 40 μg/mL GSC-derived exosomes. (B) Dose–response analysis of macrophages treated with GSC-derived exosomes.

**
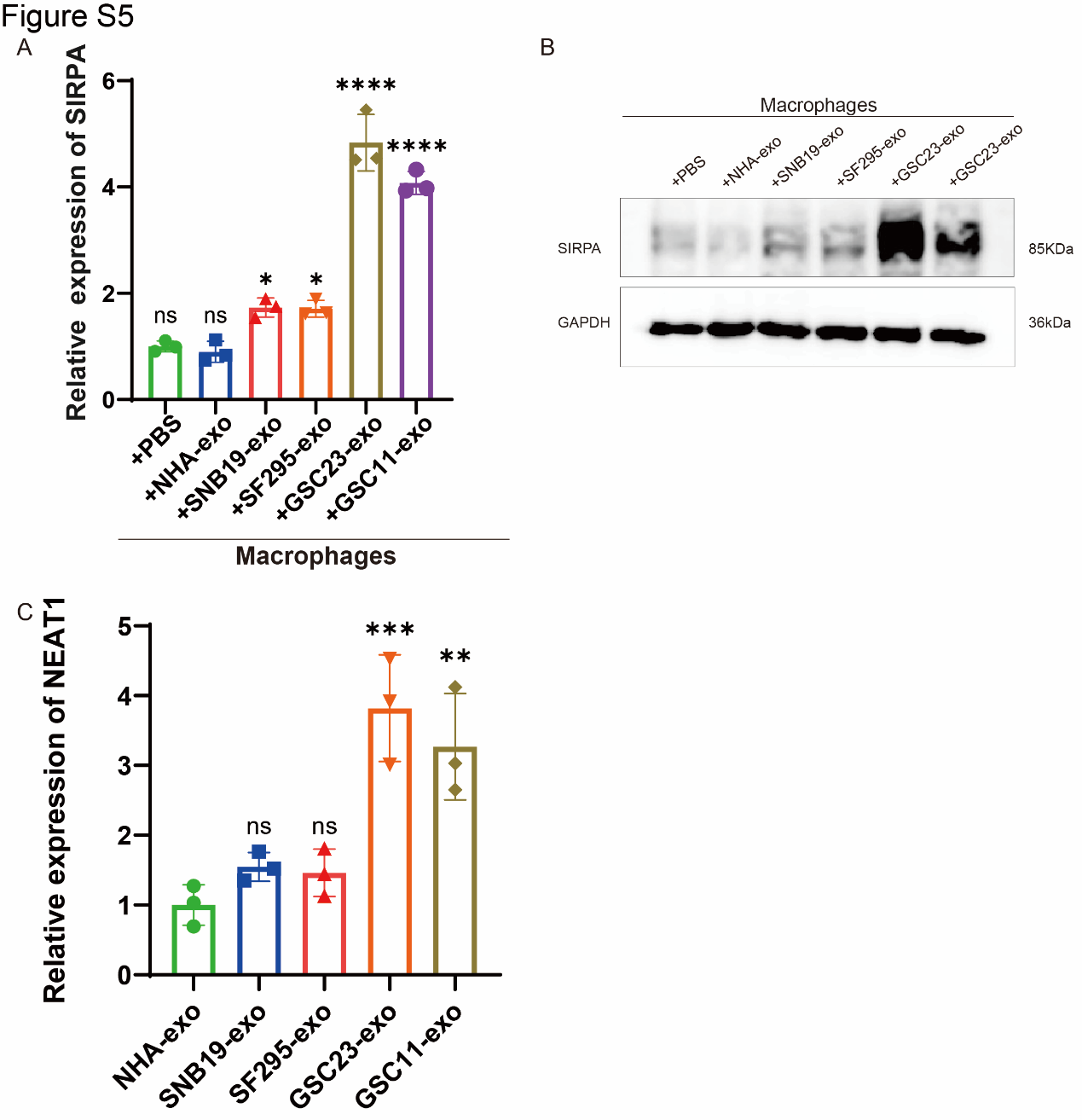
**

**FIG. S5. GSC-derived exosomes induced SIRPA^+^ macrophages.** (A) qPCR and (B) Western blot analyses of SIRPA expression in macrophages following induction with exosomes derived from GSCs, NHAs, or glioblastoma cells (SNB19 and SF295). (C) NEAT1 expression levels in exosomes derived from GSCs, NHAs and glioblastoma cell lines were assessed by qPCR.


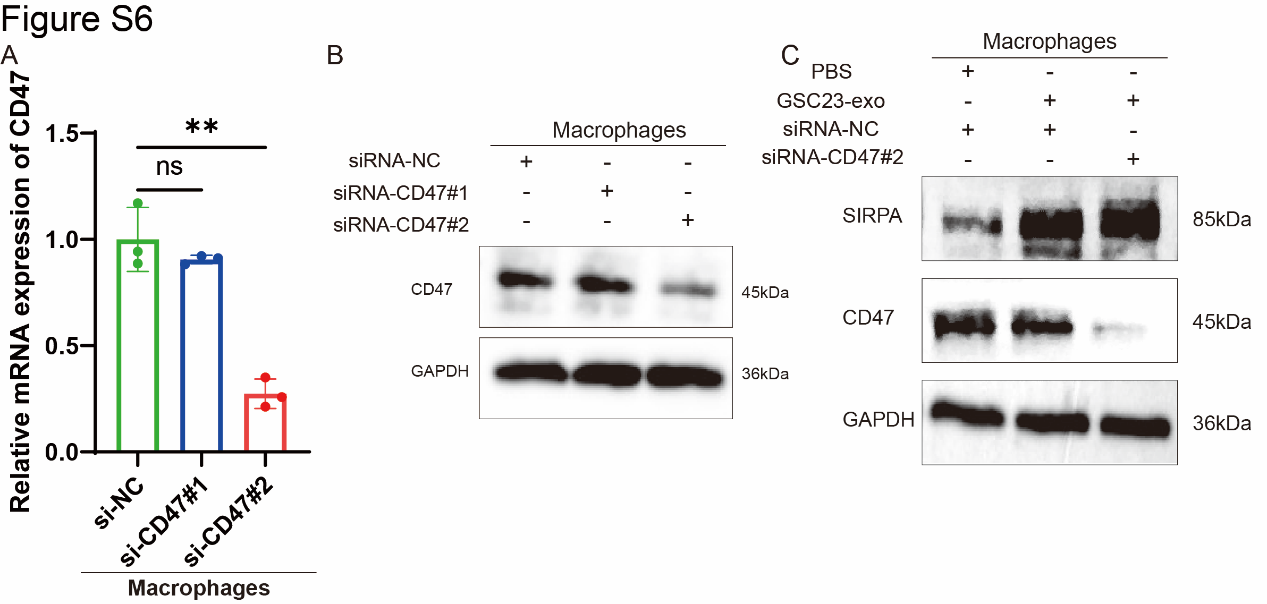


**FIG. S6. GSC-derived exosomes induced SIRPA expression in macrophages independently of macrophage CD47 expression.** (A-B) Western blot and qPCR analyses confirmed the efficiency of CD47 silencing in macrophages after siRNA transfection. (C) Western blot analysis confirmed that GSC-derived exosomes induced high SIRPA expression in macrophages independently of macrophage CD47 expression.

**
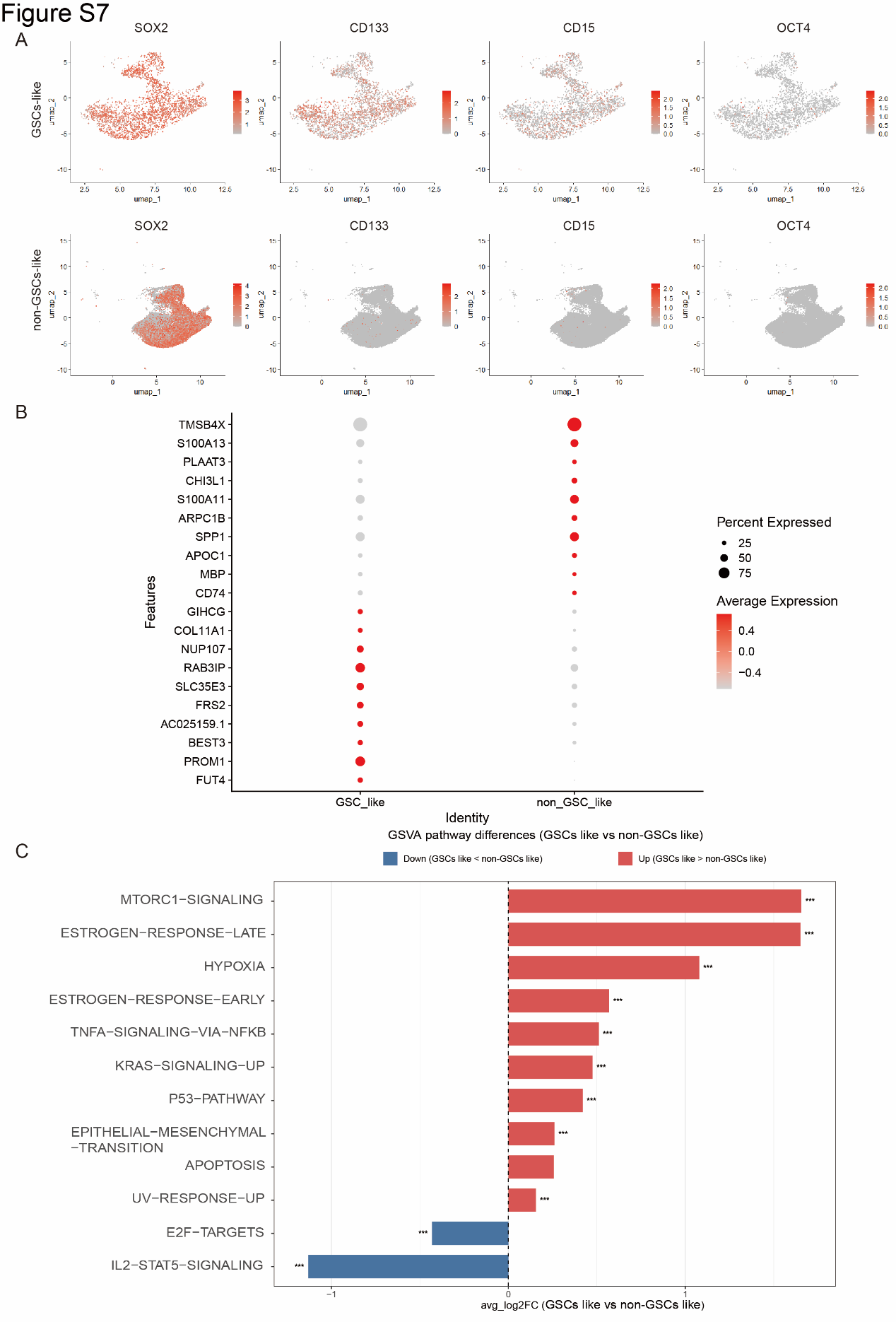
**

**FIG. S7. Different molecular characteristics between GSCs and non-GSCs tumor cells.** (A) UMAP plot displaying differential expression of tumor stem cell markers SOX2, CD133, CD15, and OCT4 in GSCs and non-GSCs tumor cells. (B) Dot plot highlighting the most significantly different genes between GSCs and non-GSCs tumor cells. (C) GSVA enrichment analysis comparing the functional differences between GSCs and non-GSCs tumor cells.


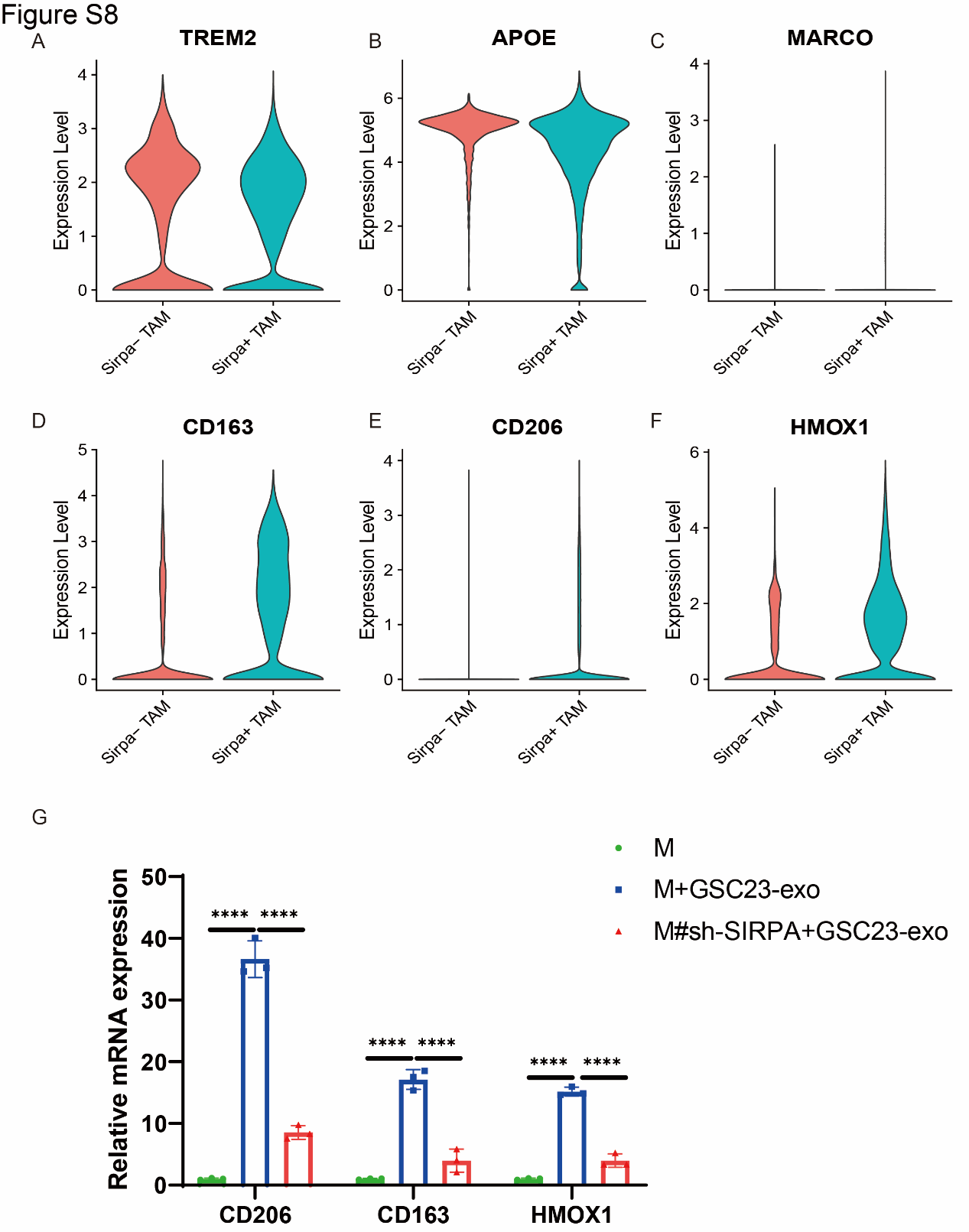


**FIG. S8. SIRPA may contribute to driving macrophages towards immunosuppressive macrophage state, characterized by high expression of CD163, CD206, and HMOX1.** (A-F) Violin plots to disclose expression difference of TREM2, APOE, MARCO, CD163, CD206, and HMOX1 both in SIRPA⁺ and SIRPA⁻ TAMs. (G) qPCR analysis to evaluate the expression of CD163, CD206, and HMOX1 in SIRPA⁺ macrophages.


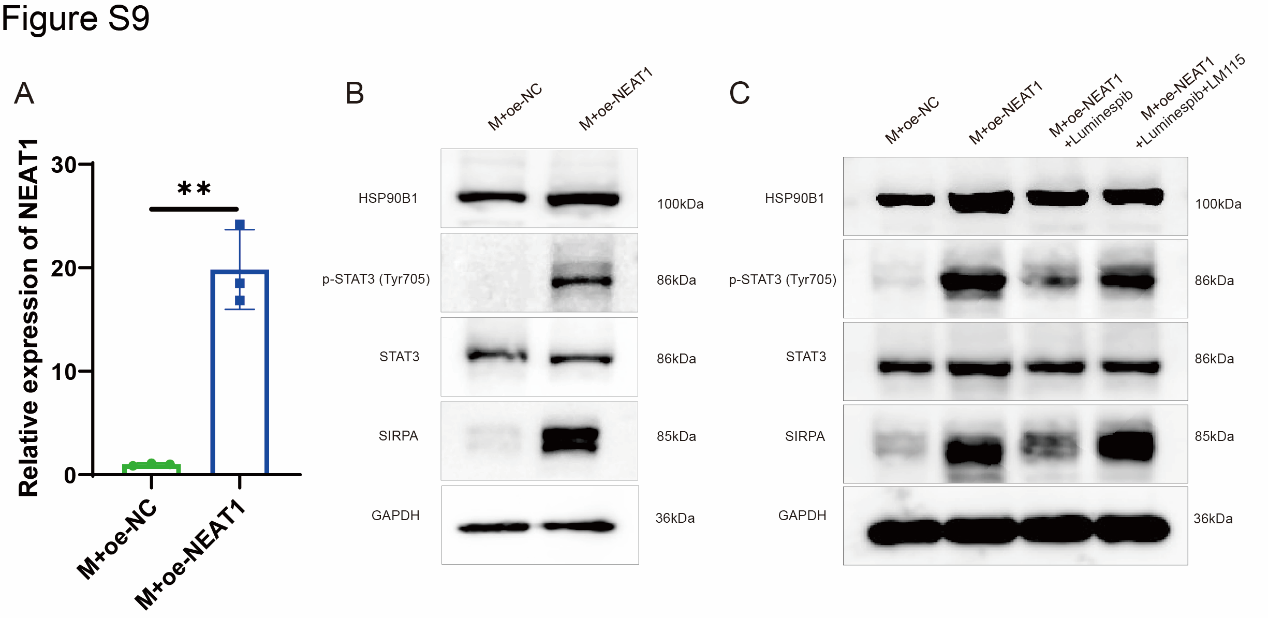


**FIG. S9. LncRNA NEAT1 induced SIRPA⁺ macrophages through the HSP90B1/STAT3 axis.** (A) qPCR verification of NEAT1 overexpression efficiency in macrophages after NEAT1 expression vector transfection. (B) Western blot analysis of SIRPA, p-STAT3, and HSP90B1 expression in macrophages after NEAT1 overexpression. (C) Western blot analysis of SIRPA, p-STAT3, and HSP90B1 expression in macrophages treated with Luminespib (HSP90B1 inhibitor) after NEAT1 overexpression, with or without additional LM115 (STAT3 activator) exposure.

**
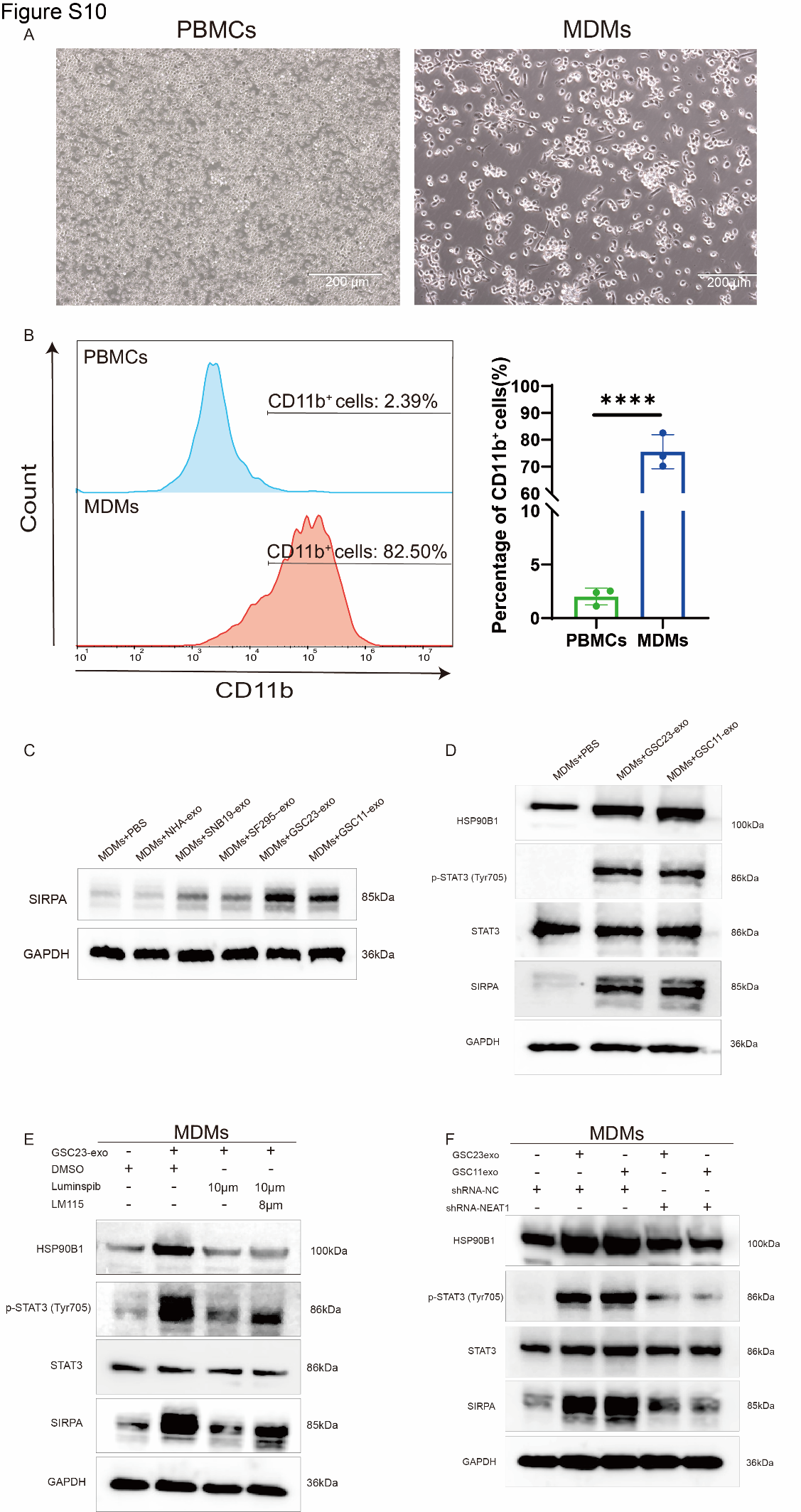
**

**FIG. S10. GSC-derived exosomal NEAT1 induces the conversion of MDMs into SIRPA⁺ macrophages via upregulating the HSP90B1-STAT3 axis.** (A) Microscopic view of isolated PBMCs and MDMs derived from patient whole blood. (B) Flow cytometry analysis of CD11b expression in PBMCs and MDMs. (C) Western blot analysis of SIRPA expression in MDMs treated with exosomes derived from different cells. (D) Western blot analysis of SIRPA, STAT3, p-STAT3, and HSP90B1 expression in MDMs after treatment with GSC-derived exosomes. (E) Western blot analysis of SIRPA, STAT3, p-STAT3, and HSP90B1 expression in MDMs after treatment with GSC-derived exosomes alone, GSC-derived exosomes plus Luminespib, or GSC-derived exosomes plus Luminespib and LM115. (F) Western blot analysis of SIRPA, STAT3, p-STAT3, and HSP90B1 expression in MDMs after treatment with GSC-derived exosomes alone or GSC-derived exosomes with NEAT1 knockdown.


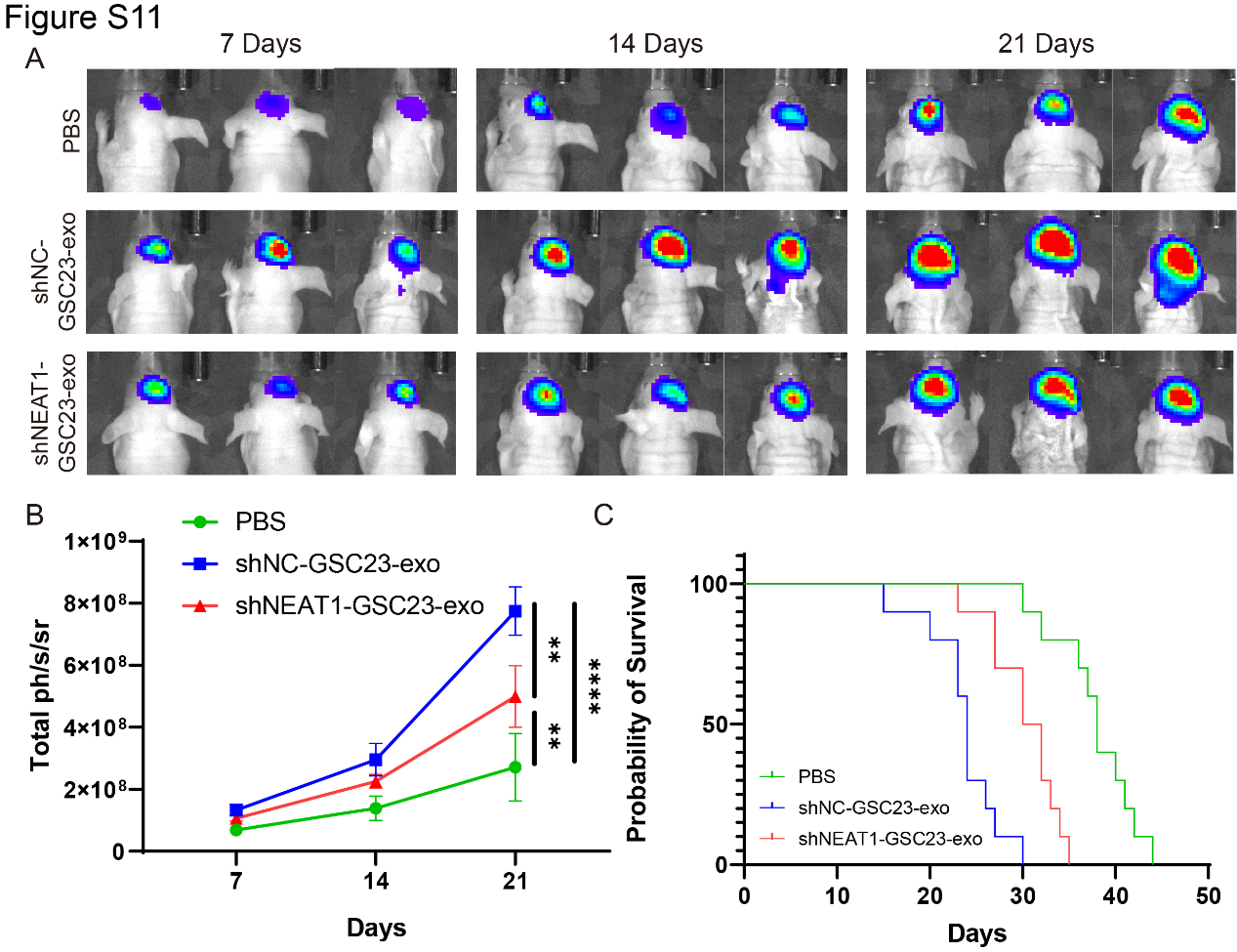


**FIG. S11. NEAT1 from GSC-derived exosomes promoted intracranial tumor progression.** (A) Bioluminescence images and (B) comparison of fluorescence intensity of intracranial xenografts established by co-inoculation of U87MG cells and macrophages together with either PBS, or shNC-GSC23 exosomes, or shNEAT1-GSC23 exosomes. (C) Survival curves of tumor-bearing mice co-inoculated with either PBS, or shNC-GSC23 exosomes, or shNEAT1-GSC23 exosomes, respectively.

**Supplementary Table 1. Sequence of primers for qRT-PCR analysis**

| **Primers used in q-PCR analysis** | | |
| --- | --- | --- |
| GAPDH | Forward primer | CAGGAGGCATTGCTGATGAT |
|  | Reverse primer | GAAGGCTGGGGCTCATTT |
| SIRPA | Forward primer | CCGCCGCTGAGAACACTGG |
|  | Reverse primer | CTTCTGTCTGATTCGGACGAGGTAG |
| CXCL8 | Forward primer | ACTGAGAGTGATTGAGAGTGGAC |
|  | Reverse primer | AACCCTCTGCACCCAGTTTTC |
| TGFBI | Forward primer | CGAGGACACCTTTGAGACCC |
|  | Reverse primer | AGGGATCTTCTCGAAGGCCT |
| IL1β | Forward primer | CCACCTCCAGGGACAGGATA |
|  | Reverse primer | TCAACACGCAGGACAGGTAC |
| HSP90B1 | Forward primer | CCAGTTTGGTGTCGGTTTCTAT |
|  | Reverse primer | CTGGGTATCGTTGTTGTGTTTTG |
| NEAT1 | Forward primer | AGTTAGCGACAGGGAGGGAT |
|  | Reverse primer | ATCTCTCCCTGTCTGTCCCC |
| CD206 | Forward primer | GGACGTGGCTGTGGATAAAT |
|  | Reverse primer | ACCCAGAAGACGCATGTAAAG |
| CD163 | Forward primer | ATCAACCCTGCATCTTTAGACA |
|  | Reverse primer | CTTGTTGTCACATGTGATCCAG |
| HMOX1 | Forward primer | GGGTGATAGAAGAGGCCAAGA |
|  | Reverse primer | AGCTCCTGCAACTCCTCAAA |

**Supplementary Table 2. Primary antibodies used in the study**

| **Antibody** | **Company (Cat. No.)** | **Working dilutions** |
| --- | --- | --- |
| SIRPA | CST (13379) | WB:1/1000, IF:1/1000 |
| CD133 | CST (64326) | WB:1/1000, IF:1/1000 |
| SOX2 | CST (3579) | WB:1/1000 |
| CD11b | CST (93169) | WB:1/1000 |
| CD9 | Abcam (ab263019) | WB:1/1000 |
| TSG101 | Abcam (ab125011) | WB:1/1000 |
| Calnexin | Abcam (ab133615) | WB:1/1000 |
| E-Cadherin | CST (3195) | WB:1/1000, IHC:1/200 |
| N-Cadherin | CST (13116) | WB:1/1000, IHC:1/200 |
| Vimentin | CST (5741) | WB:1/1000, IHC:1/200 |
| CXCL8 | Boster (PB0685) | WB:1/1000 |
| STAT3 | CST (9139) | WB:1/1000, IP: 2 ug for 2 mg of total protein lysate |
| p-STAT3 | CST (9145) | WB:1/1000 |
| HSP90B1 | Proteintech (14700-1-AP) | WB:1/2000, IP: 2 ug for 2 mg of total protein lysate |
| Ubiquitin | CST (43124) | WB:1/5000 |
| GAPDH | CST (97166) | WB:1/10000 |
| Rabbit (DA1E) Monoclonal Antibody IgG Isotype Control | CST (3900) | IP: 2 ug for 2 mg of total protein lysate |
| **Secondary Antibody** | | |
| Anti-rabbit IgG | CST (7074) | WB:1/5000 |
| Anti-mouse IgG | CST (7076) | WB:1/5000 |
